# Subphenotype-specific serum proteomic signatures in patients with ARDS

**DOI:** 10.1186/s40560-026-00951-x

**Published:** 2026-10-01

**Authors:** Michihito Kyo, Masashi Kawami, Yasuo Uchida, Kazuya Kikutani, Daisuke Kasugai, Shinichiro Ohshimo, Nobuyuki Hirohashi, Nobuaki Shime

**Affiliations:** 1 Department of Emergency and Critical Care Medicine, Graduate School of Biomedical and Health Sciences, Hiroshima University, 1-2-3 Kasumi, Minami-Ku, Hiroshima, 734-8551 Japan; 2 Department of Radiation Disaster Medicine, Research Institute for Radiation Biology and Medicine, Hiroshima University, Hiroshima, Japan; 3 Department of Pharmaceutics, School of Pharmaceutical Sciences, Mukogawa Women’s University, Nishinomiya, Japan; 4 Department of Molecular Systems Pharmaceutics, Graduate School of Biomedical and Health Sciences, Hiroshima University, Hiroshima, Japan; 5 Department of Emergency and Critical Care Medicine, Nagoya University Graduate School of Medicine, Nagoya, Japan; 6 Institutes of Innovation for Future Society, Institute of Nano-Life-Systems, Nagoya University, Nagoya, Japan

**Keywords:** ARDS, Myeloid, Proteome, Subphenotype

## Abstract

**Background:**

Acute respiratory distress syndrome (ARDS) is associated with a substantial burden of morbidity and mortality. Previous studies have identified reproducible hyperinflammatory and hypoinflammatory ARDS subphenotypes with distinct clinical outcomes and treatment responses. Although recent transcriptomic studies have provided insights into biological differences between these subphenotypes, the role of the circulating proteome remains incompletely understood.

**Methods:**

In this prospective study, patients with ARDS were classified into hyperinflammatory or hypoinflammatory subphenotypes using a previously validated 3-variable classifier based on bicarbonate, interleukin-6, and soluble tumor necrosis factor receptor 1. Serum proteomic profiling was performed to identify differentially abundant proteins between subphenotypes. Biological pathways were examined using gene set enrichment analysis. To provide cellular expression context for the subphenotype-associated serum proteins, serum proteomic data were mapped onto publicly available single-cell RNA-seq datasets from peripheral blood mononuclear cells, neutrophils, and tracheal aspirates.

**Results:**

Among 31 patients with ARDS, 13 (42%) were classified as having the hyperinflammatory subphenotype and 18 (58%) as having the hypoinflammatory subphenotype. Both 28-day and in-hospital mortality were higher in the hyperinflammatory subphenotype than in the hypoinflammatory subphenotype (46% vs. 11%, P=0.04; 62% vs. 11%, P=0.01, respectively). In total, 54 serum proteins were differentially abundant between subphenotypes. These proteins represented biologically relevant categories, including endothelial injury, immunoregulation, coagulation/fibrinolysis, complement regulation, tissue remodeling, and metabolism-related proteins. Gene set enrichment analysis identified pathways differentially enriched between subphenotypes, including cell adhesion, cell–cell signaling, and cytokine responses (all FDR < 0.10). In external single-cell RNA-seq reference datasets, genes encoding the subphenotype-associated proteins were expressed predominantly in CD14 and CD16 monocytes in PBMCs, showed distinct expression patterns across immature and CD163+IL1R2^hi^ circulating neutrophil states, and were expressed mainly in macrophage and monocyte populations, with additional epithelial expression, in tracheal aspirates.

**Conclusions:**

Serum proteomic profiling identified distinct protein patterns associated with established ARDS inflammatory subphenotypes, providing insights into the biological heterogeneity of ARDS.

**Supplementary Information:**

The online version contains supplementary material available at https://doi.org/10.1186/s40560-026-00951-x.

## Introduction

Acute respiratory distress syndrome (ARDS) is a clinical syndrome of acute hypoxemic respiratory failure caused by diffuse lung inflammation and edema [1]. Despite advances in supportive care, ARDS remains associated with substantial morbidity and mortality, with reported mortality rates of approximately 40% [2]. Increasing evidence suggests that ARDS is not a single biological entity but a heterogeneous syndrome comprising distinct subphenotypes with different inflammatory profiles, clinical outcomes, and potentially differential responses to therapy [3–7]. However, the biological mechanisms underlying ARDS subphenotypes remain incompletely understood.

Among the various pathobiological pathways involved in ARDS, proteins play essential roles as mediators, biomarkers, and potential therapeutic targets [8, 9]. Circulating serum proteins reflect diverse biological processes relevant to ARDS, including epithelial and endothelial injury, inflammation, and coagulation [1]. Although recent transcriptomic studies have advanced our understanding of cellular programs associated with ARDS subphenotypes [10, 11], mRNA profiles do not necessarily capture protein abundance, post-transcriptional regulation, or circulating effector molecules [12]. Thus, proteomic profiling may provide complementary and clinically relevant insights into the biological mechanisms underlying ARDS subphenotypes. However, despite its biological and translational relevance, studies applying serum proteomics to delineate the pathobiology of established ARDS subphenotypes remain limited.

To address this knowledge gap, we investigated the serum proteome signature associated with ARDS subphenotypes. We hypothesized that serum proteomic profiling would reveal biological pathways underlying ARDS subphenotypes and provide mechanistic insights relevant to precision medicine in ARDS.

## Methods

### Study design, setting, and data collection

We studied adult patients with ARDS who received mechanical ventilation in the ICU of Hiroshima University Hospital between March 2016 and February 2018 and had residual serum samples available for proteomic analysis. ARDS was clinically defined according to the Berlin criteria [13]. Patients younger than 18 years were excluded. The study protocol was approved by the Institutional Review Board of Hiroshima University Hospital (approval number: E−447, approved on August 5, 2016). The requirement for informed consent was waived because this was an observational study using residual blood samples obtained for clinical purposes. We collected demographic and clinical data, including age, sex, underlying clinical conditions, laboratory data, and Sequential Organ Failure Assessment (SOFA) and Acute Physiology and Chronic Health Evaluation (APACHE) II scores at ICU admission. We also recorded the treatments during the ICU stay and in-hospital mortality.

### Sample collection and serum biomarker measurement

Blood samples were collected from patients within 24 h after ARDS diagnosis and from healthy research volunteers recruited at Hiroshima University Hospital. For both groups, blood samples were centrifuged, and serum was immediately stored at − 80 °C until analysis. In patients with ARDS, interleukin-6 (IL-6) and soluble tumor necrosis factor receptor 1 (sTNFR1) concentrations were measured using an enzyme-linked immunosorbent assay (R&D Systems, Inc., MN, USA) following the manufacturer’s protocol.

### ARDS subphenotype assignment

ARDS subphenotypes (e.g., hyper/hypoinflammatory subphenotype) were determined by a previously validated 3-variable classifier model based on IL-6, sTNFR1, and bicarbonate [7, 14]. Patients with a predicted probability of hyperinflammatory subphenotype assignment >0.5 were classified as having the hyperinflammatory subphenotype; the remaining patients were classified as having the hypoinflammatory subphenotype.

### Serum proteomic profiling

Serum samples were diluted 5-fold and processed using a urea-based protein extraction method [15]. In brief, proteins were denatured in 8 M urea/0.1 M Tris–HCl (pH 8.5), reduced with 20 mg/mL dithiothreitol for 60 min at room temperature, and alkylated with 50 mg/mL iodoacetamide for 60 min at room temperature in the dark. After dilution of urea to 1.2 M with 0.1 M Tris–HCl (pH 8.5), proteins were digested with lysyl endopeptidase (Lys-C, enzyme:protein ratio 1:100, w/w) at 30 °C for 3 h, followed by trypsin digestion (enzyme:protein ratio 1:100, w/w) at 37 °C for 16 h. The resulting peptides were acidified with trifluoroacetic acid and desalted using Empore SDB-XD stage tips. Purified peptides were dried, reconstituted in 2% acetonitrile/0.1% formic acid. The cleaned peptide samples (0.25 μg peptide) were injected into a Vanquish Neo UHPLC system (Thermo Fisher Scientific, Waltham, MA, USA) coupled with a ZenoTOF 7600 mass spectrometer (SCIEX, Framingham, MA, USA), which was set up for a Trap and Elute injection and analyzed by ZenoSWATH-MS acquisition as described previously [16]. Protein identification and quantification of SWATH data were performed by library-free search using human FASTA (one protein sequence per gene) and DIA-NN version 2.0.2 [17] with DIA-NN default settings.

### Statistical analyses

ARDS subphenotypes were assigned using the 3-variable classifier model based on IL-6, sTNFR1, and bicarbonate, as described above. Proteomic data were preprocessed by excluding proteins that could not be mapped to gene symbols and proteins with more than 30% missing values across all samples. Missing values among the retained proteins were imputed using the *missForest* R package, and protein abundance values were subsequently log2 transformed. Differentially abundant proteins between the hyperinflammatory and hypoinflammatory subphenotypes were identified using the *limma* R package [18]. Differentially abundant proteins were defined as proteins with a false discovery rate (FDR) <0.10 and an absolute log2 fold change ≥0.59, corresponding to an approximately 1.5-fold difference. Comparisons with healthy controls were summarized descriptively using log2 fold changes and concordance in direction across the hyperinflammatory–control, hypoinflammatory–control, and ARDS–control comparisons. An exploratory analysis of differentially abundant proteins between 28-day survivors and non-survivors was performed in the overall ARDS cohort using limma with adjustment for APACHE II score. To characterize biological pathways associated with ARDS subphenotypes, gene set enrichment analysis (GSEA) using the ranked list of all quantified proteins was performed using the *fgsea* R package [19] with Gene Ontology biological process gene sets and MSigDB HALLMARK gene sets. For visualization of Gene Ontology results, significant terms were restricted to a minimum ontology depth of four and reduced using Wang semantic similarity with a cutoff of 0.7. To provide external cellular expression context for the differentially abundant proteins, we mapped differentially abundant proteins to publicly available single-cell RNA-seq datasets from peripheral blood mononuclear cell (PBMC), whole-blood, and lower respiratory tract samples (GEO: GSE151263 [20]; GEO: GSE157789 [21]; GEO: GSE200848 [22]). For GSE157789, the analysis was restricted to circulating neutrophils collected within 72 h of ICU admission from patients with non-COVID bacterial ARDS and used the cell-state annotations provided by the original investigators. Detailed dataset and cohort characteristics are provided in Table S1. Within each dataset, normalized expression was averaged across cells within each annotated cell type, standardized to Z-scores for each gene across cell types, and visualized as heatmaps. We performed several sensitivity analyses. First, differentially abundant proteins between the ARDS subphenotypes were re-evaluated with adjustment for APACHE II score. Second, we excluded proteins showing a nominally significant Spearman correlation with either clinical IL-6 or sTNFR1 (P <0.05) and repeated GO Biological Process GSEA using the remaining proteins. We conducted the statistical analysis by using R version 4.4.1 (R Foundation, Vienna, Austria). We accounted for multiple testing using the Benjamini–Hochberg FDR method that allows for the interpretation of statistical significance in the context of multiple hypothesis testing [23].

## Results

### Patient characteristics and outcomes

A total of 31 patients with ARDS and 5 healthy controls underwent proteomic profiling. The age and sex distributions of patients with ARDS and healthy controls are summarized descriptively in Table S2. Overall, the median age was 67 years (interquartile range [IQR] 59–76), and 22 patients (71%) were male (Table 1). The median SOFA and APACHE II scores were 9 and 28, respectively. According to the Berlin definition, 5 patients (16%) had mild ARDS, 13 (42%) had moderate ARDS, and 13 (42%) had severe ARDS. The median interval from onset of the ARDS risk factor to the ARDS onset was 2 days (IQR, 0–4 days). The risk-factor onset and ARDS onset occurred on the same calendar day in 9 of 31 patients (29%). Based on the 3-variable classifier model using IL-6, sTNFR1, and bicarbonate, 13 patients were classified as having the hyperinflammatory subphenotype and 18 patients as having the hypoinflammatory subphenotype. Patients with the hyperinflammatory subphenotype had lower BMI and bicarbonate levels and higher SOFA and APACHE II scores than those with the hypoinflammatory subphenotype (all *P* <0.05). Overall, 8 patients (26%) died within 28 days and 10 (32%) died during hospitalization. Both 28-day mortality and in-hospital mortality were significantly higher in the hyperinflammatory subphenotype than in the hypoinflammatory subphenotype (46% vs. 11%, *P*=0.04; and 62% vs. 11%, *P*=0.01, respectively) (Table 2).

**Table 1:** Baseline characteristics of patients with ARDS stratified by the subphenotype

	**Total** **(N=31)**	**Hyperinflammatory subphenotype** **(N=13)**	**Hypoinflammatory subphenotype** **(N=18)**	**P value**
Age (years), median (IQR)	67 (59–76)	67 (63–72)	67 (58–76)	0.69
Male sex	22 (71)	8 (62)	14 (78)	0.43
BMI (kg/m^2^), median (IQR)	23 (20–25)	20 (19–23)	25 (23–27)	0.01
Asian race	31 (100)	13 (100)	18 (100)	1.00
**Coexisting conditions**				
Chronic heart disease	1 (3)	0 (0)	1 (6)	1.00
Chronic respiratory disease	4 (13)	1 (8)	3 (17)	0.62
Liver failure	8 (26)	2 (15)	1 (6)	0.56
Chronic kidney disease	3 (10)	1 (8)	7 (39)	0.10
Immunodeficiency	5 (16)	2 (15)	3 (17)	1.00
**ARDS characteristics**				
Cause of ARDS				0.81
Pneumonia	22 (71)	9 (69)	13 (72)	
Aspiration	1 (3)	1 (8)	0 (0)	
Sepsis	6 (19)	2 (15)	4 (22)	
Other	2 (7)	1 (8)	1 (6)	
Direct ARDS	24 (77)	11 (85)	13 (72)	0.67
**Laboratory data on ICU admission**				
White blood cell (/μL), median (IQR)	10,910 (4,585–12,495)	7,700 (3,640–10,960)	11,435 (6,978–14,455)	0.16
Platelet (× 10^3^/μL), median (IQR)	148 (74–208)	171 (78–192)	147 (73–226)	0.73
Bicarbonate (mmol/L), median (IQR)	24 (20–27)	19 (16–20)	26 (24–29)	<0.001
Creatinine (mg/dL), median (IQR)	1.0 (0.6–2.2)	2.2 (1.6–4.1)	0.8 (0.6–1.0)	0.002
IL-6 (pg/mL), median (IQR)	509 (132–1283)	3,423 (567–5,044)	203 (44–491)	<0.001
sTNFR1 (pg/mL), median (IQR)	7,283 (4,339–11,295)	12,480 (8,891–22,964)	4,687 (3,275–7,230)	<0.001
**Severity and ventilation settings**				
SOFA score	9 (7–11)	12 (10–14)	8 (6–10)	0.001
APACHE II score	28 (22–33)	33 (30–35)	23 (18–28)	<0.001
ARDS grade (Berlin definition)				0.12
Mild	5 (16)	0 (0)	5 (28)	
Moderate	13 (42)	6 (46)	7 (39)	
Severe	13 (42)	7 (54)	6 (33)	
PaO_2_/F_I_O_2_, median (IQR)	119 (86–179)	119 (80–186)	137 (89–172)	0.49
PEEP, median (IQR)	12 (10–12)	12 (10–15)	12 (9–12)	0.12
Driving pressure, median (IQR)	13 (10–16)	14 (12–17)	12 (10–16)	0.27
**Therapy for ARDS**				
Neuromuscular blockage	6 (19)	1 (8)	5 (28)	0.36
Prone positioning	3 (10)	2 (15)	1 (6)	0.56
V-V ECMO	5 (16)	2 (15)	3 (17)	1.00
Steroid therapy	16 (52)	8 (62)	8 (44)	0.47

Data are presented as median (interquartile range) or N (%). Variables were compared using Mann–Whitney U test or Fisher's exact as appropriate

APACHE, Acute Physiology and Chronic Health Evaluation; ARDS, Acute Respiratory Distress Syndrome; BMI, body mass index; V-V ECMO, veno-venous extracorporeal membrane oxygenation; ICU, intensive care unit; IL-6, interleukin 6; IQR, interquartile range; PEEP, positive end-expiratory pressure; PF, PaO_2_/F_I_O_2_; SOFA, Sequential Organ Failure Assessment; sTNFR1, soluble tumor necrosis factor receptor-1

**Table 2:** Outcomes of patients with ARDS stratified by the subphenotype

	**Total** **(N=31)**	**Hyperinflammatory subphenotype** **(N=13)**	**Hypoinflammatory subphenotype** **(N=18)**	**P value**
28-day mortality	8 (26)	6 (46)	2 (11)	0.04
In-hospital mortality	10 (32)	8 (62)	2 (11)	0.01
Length-of-ICU stay, median (IQR)	15 (7–22)	15 (7–23)	15 (8–19)	0.94
Tracheostomy	12 (39)	6 (46)	6 (33)	0.71

Data are presented as median (interquartile range) or N (%). Variables were compared using Mann–Whitney U test or Fisher's exact test as appropriate

ICU, intensive care unit; IQR, interquartile range

### Subphenotype-specific proteins and pathways

**Fig. 1:**
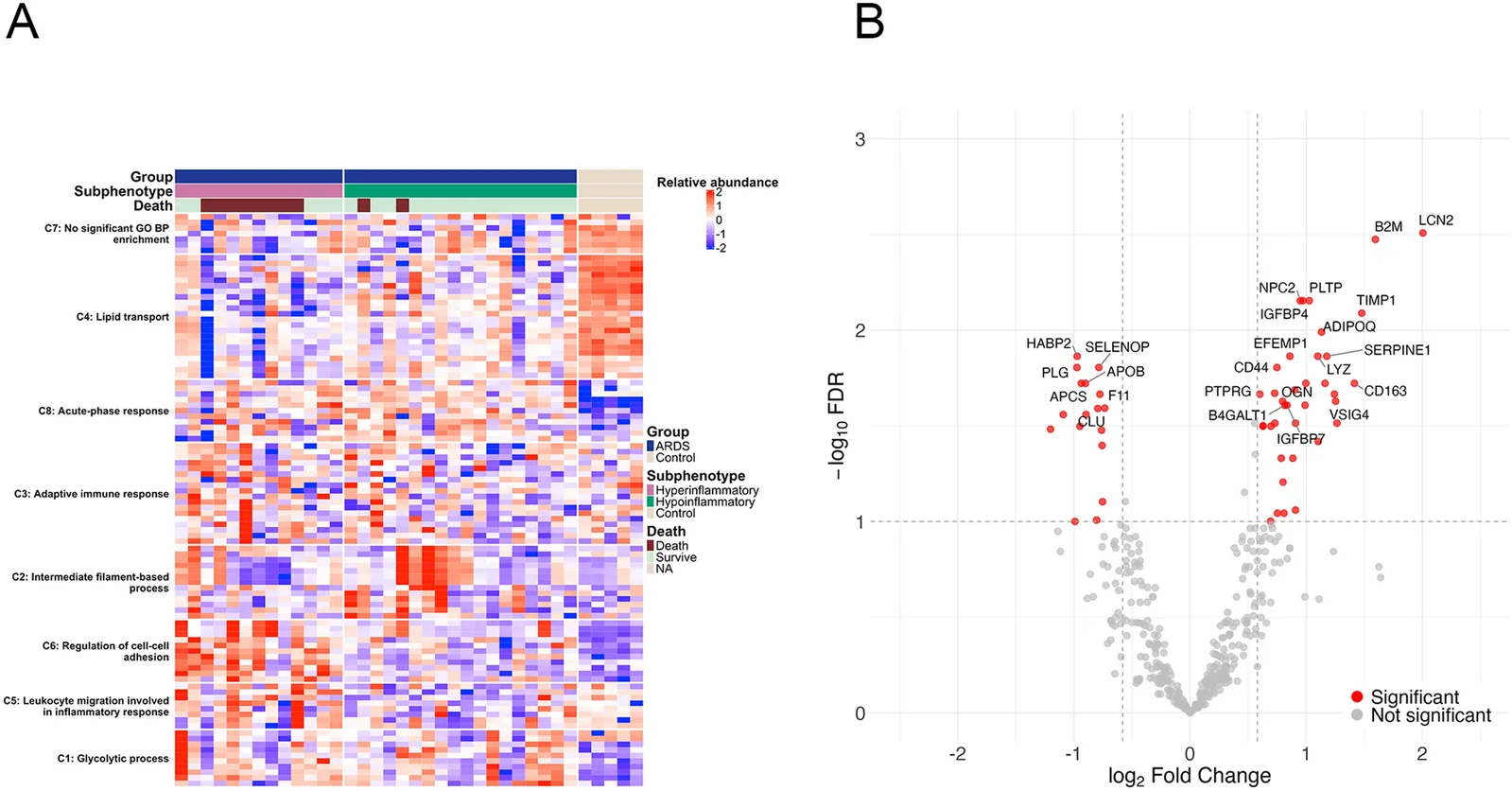
Serum proteomic signatures associated with ARDS subphenotypes. **A** Heatmap of the 100 proteins with the greatest variance across patients with ARDS and control subjects. Each column represents an individual subject, and each row represents a protein. Protein abundance values were standardized across subjects, with red and blue indicating higher and lower relative abundance, respectively. Proteins were separated into eight data-driven clusters (C1–C8) using k-means clustering. Representative significantly enriched Gene Ontology Biological Process terms are shown for each cluster: glycolytic process (C1), intermediate filament-based process (C2), adaptive immune response (C3), lipid transport (C4), leukocyte migration involved in inflammatory response (C5), regulation of cell–cell adhesion (C6), and acute-phase response (C8); C7 showed no significant enrichment. Clinical annotations, including study group, inflammatory subphenotype, and survival status, are shown above the heatmap. **B** Volcano plot comparing serum protein abundance between hyperinflammatory and hypoinflammatory ARDS subphenotypes. The x-axis shows the log₂ fold change, and the y-axis shows the −log₁₀ FDR. Positive log₂ fold changes indicate higher abundance in the hyperinflammatory subphenotype, whereas negative values indicate higher abundance in the hypoinflammatory subphenotype. Significantly differentially abundant proteins, defined as FDR < 0.10 and |log₂ fold change|≥ 0.59, are shown in red, and non-significant proteins are shown in gray. Abbreviations: ARDS, acute respiratory distress syndrome; FDR, false discovery rate

**Fig. 2:**
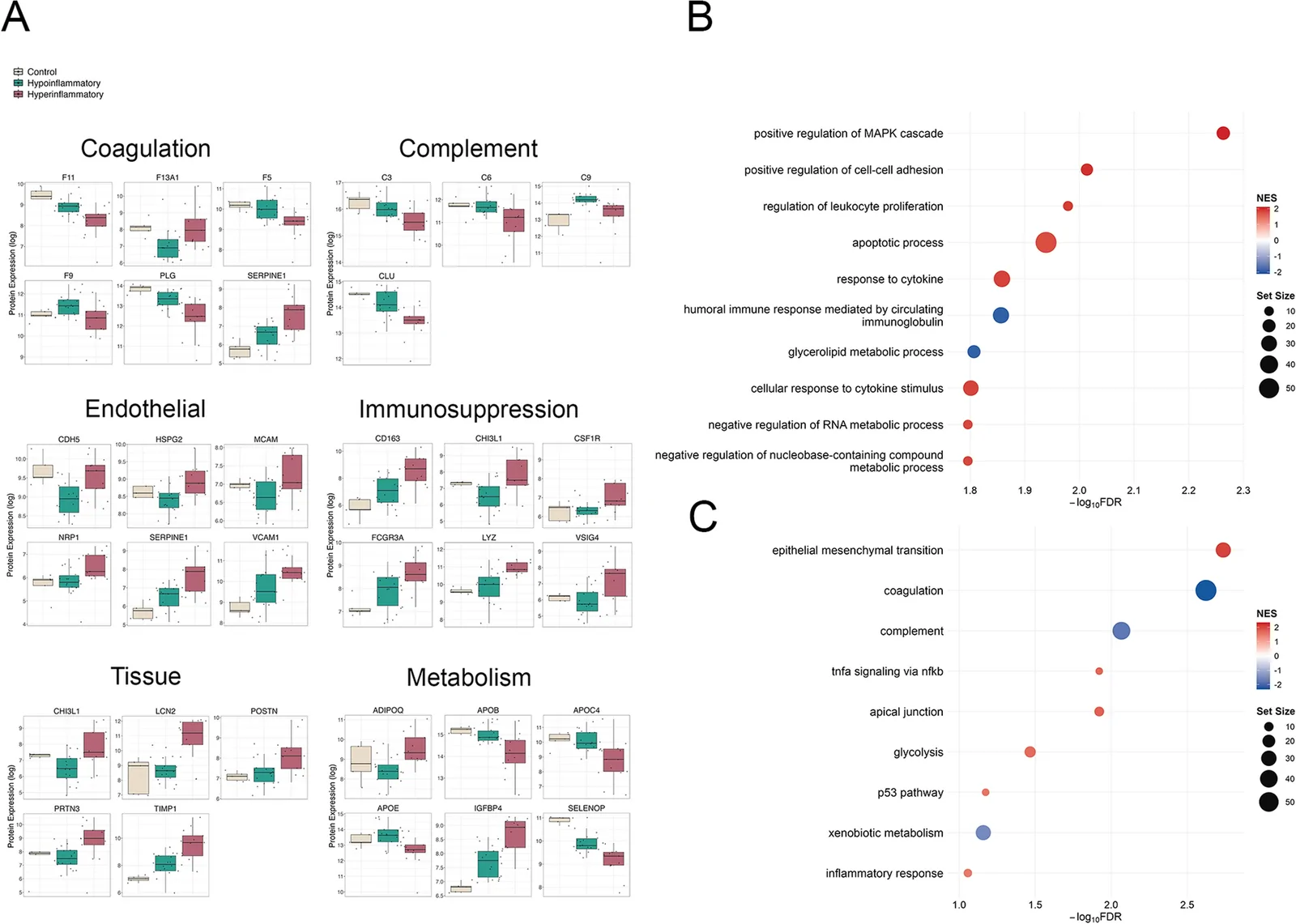
Biological pathways and representative serum proteins associated with ARDS subphenotypes. **A** Boxplots showing the abundance of representative serum proteins grouped by biological processes, including endothelial injury, immunoregulation, coagulation, complement, tissue remodeling, and metabolic dysregulation. Protein abundance is shown on a log_2_ scale. Colors indicate hyperinflammatory and hypoinflammatory ARDS subphenotypes and control subjects. **B** Gene set enrichment analysis using Gene Ontology Biological Process gene sets. The 10 significant terms with the lowest FDR are shown after applying a minimum ontology depth of four and Wang semantic-similarity reduction with a cutoff of 0.7. The x-axis shows the −log₁₀ FDR. Dot size represents the number of proteins included in each gene set, and color indicates the normalized enrichment score. **C** Gene set enrichment analysis using MSigDB HALLMARK gene sets. Significantly enriched terms with the lowest FDR are shown. The x-axis shows the −log₁₀ FDR. Dot size represents the number of proteins included in each gene set, and color indicates the normalized enrichment score. Abbreviations: ARDS, acute respiratory distress syndrome; FDR, false discovery rate; GO, Gene Ontology; NES, normalized enrichment score

Proteomic profiling measured 861 serum proteins across patients with ARDS and healthy controls. Of the 861 proteins in the unfiltered proteomic output, three were excluded because they could not be mapped to gene symbols, and 421 were excluded because they had more than 30% missing values across all samples. Consequently, 437 proteins were retained for downstream analysis. In a descriptive comparison with healthy controls, 176 of 437 proteins showed an absolute log2 fold change ≥0.59 in the ARDS cohort (Table S3). The heatmap demonstrated distinct proteomic patterns across the hyperinflammatory and hypoinflammatory subphenotypes as well as healthy controls (Fig. 1A). In total, 54 serum proteins were differentially abundant between the hyperinflammatory and hypoinflammatory ARDS subphenotypes (Fig. 1B and Table S4). In a sensitivity analysis with adjustment for APACHE II score, 30 of the original 54 proteins remained significant, and the direction of association was unchanged for all 54 proteins (Table S5). In a separate threshold-sensitivity analysis, 46 of the 54 proteins (85%) remained significant at FDR <0.05 (Table S6). In descriptive comparisons with healthy controls, 353 of 437 proteins (80.8%) showed the same direction of change in the hyperinflammatory–control and hypoinflammatory–control comparisons (Figure S1). All 121 proteins with an absolute log2 fold change ≥0.59 in each of the hyperinflammatory–control, hypoinflammatory–control, and ARDS–control comparisons showed concordant directions (Table S7). An exploratory analysis identified eight proteins associated with 28-day mortality (Table S8). SERPINE1 overlapped with the 54 subphenotype-associated proteins. Differentially abundant proteins between subphenotypes included proteins involved in several biologically relevant categories, including endothelial injury (e.g., VCAM1, NRP1), immunoregulation (e.g., CD163, CSF1R, VSIG4), coagulation (e.g., PLG, SERPINE1), complement regulation (e.g., C3, C6, CLU), tissue remodeling (e.g., LCN2, CHI3L1, TIMP1), and metabolic dysregulation (e.g., APOB, APOC4, IGFBP4, SELENOP) (Fig. 2A). Among nine proteins reported to differ between subphenotypes in an independent serum proteomic study [24], three (PTGDS, NRP1, SERPIND1) overlapped with our 54-protein set. All three showed concordant directions between studies. GSEA identified 171 significantly enriched Gene Ontology Biological Process terms between subphenotypes (all FDR<0.10). Notably, pathways related to MAPK cascade, cell–cell adhesion, leukocyte proliferation, and cytokine response were significantly enriched (Fig. 2B). Complete results are provided in Table S9. GSEA also identified 9 differentially enriched HALLMARK terms (all FDR<0.10). Coagulation, epithelial mesenchymal transition, apical junction, and glycolysis were significantly enriched (Fig. 2C). In a sensitivity analysis, 168 of 437 proteins showing a nominally significant correlation with either clinical IL-6 or sTNFR1 were excluded. GSEA identified 65 significantly enriched GO Biological Process terms, including cell adhesion, cell–cell signaling, and cytokine response, whereas MAPK-related terms were not significantly enriched (Table S10).

### Cellular expression context in external scRNA-seq data

**Fig. 3:**
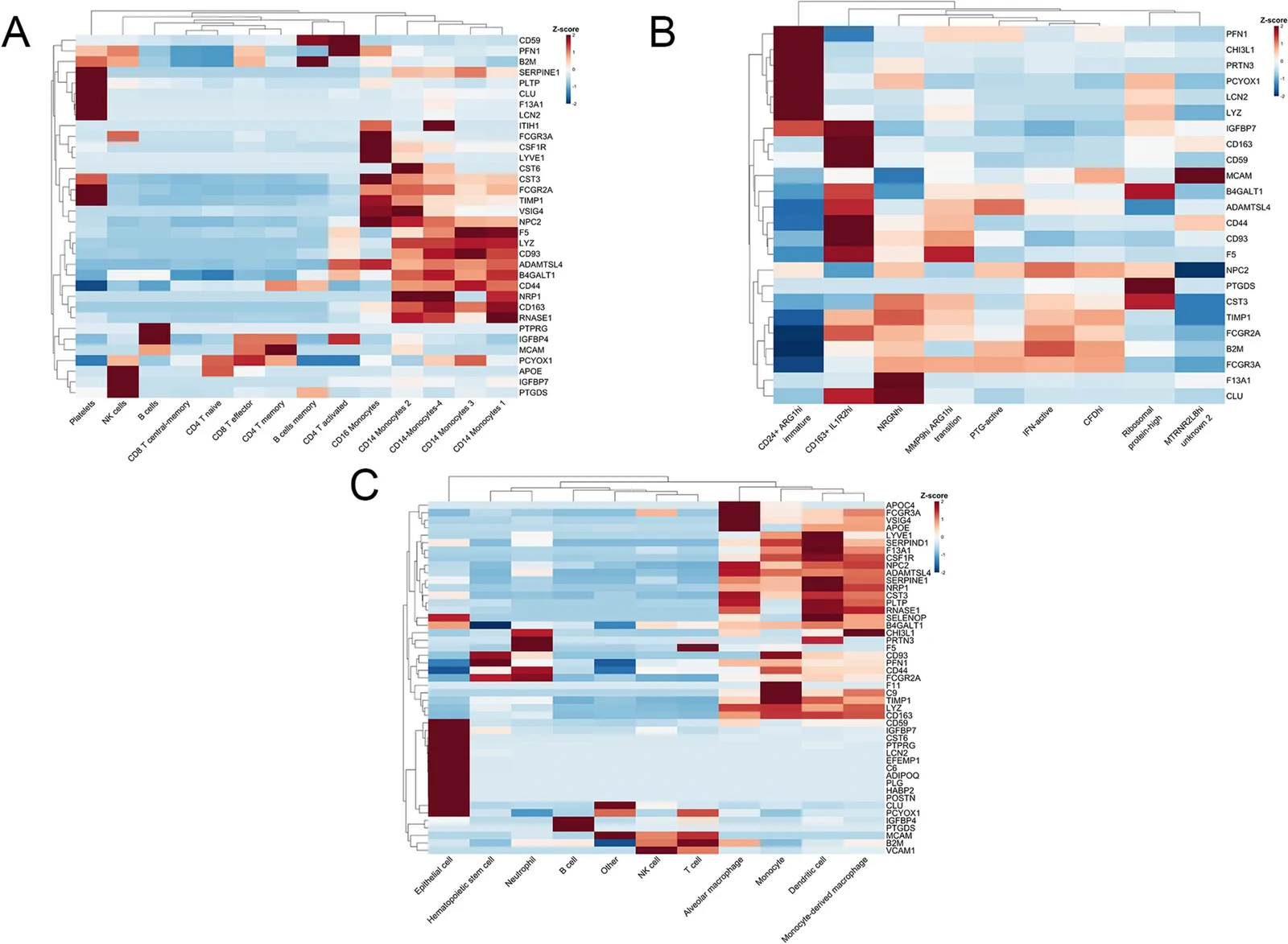
Cellular expression context of subphenotype-associated proteins in PBMC, neutrophil, and tracheal aspirate single-cell RNA-seq datasets. **A** Heatmap showing the cell-type-specific expression patterns of genes encoding differentially abundant serum proteins mapped to publicly available peripheral blood mononuclear cell single-cell RNA-sequencing data. Rows represent genes, and columns represent immune cell populations. Colors indicate scaled expression levels across cell types. **B** Heatmap showing the cell-type-specific expression patterns of genes encoding differentially abundant serum proteins mapped to publicly available neutrophil single-cell RNA-sequencing dataset of samples collected within 72 h of ICU admission from patients with non-COVID bacterial ARDS. Rows represent genes, and columns represent neutrophil populations. Colors indicate scaled expression levels across cell types. **C** Heatmap showing the cell-type-specific expression patterns of genes encoding differentially abundant serum proteins mapped to publicly available airway single-cell RNA-sequencing data. Rows represent genes, and columns represent airway and immune cell populations. Colors indicate scaled expression levels across cell types. ARDS, acute respiratory distress syndrome; PBMC, peripheral blood mononuclear cell; scRNA-seq, single-cell RNA sequencing

To provide cellular expression context for the subphenotype-associated serum proteins, we mapped differentially abundant proteins between ARDS subphenotypes onto external PBMC, circulating neutrophil, and tracheal aspirate scRNA-seq data (Fig. 3A–C). In PBMCs, genes encoding many mapped proteins were preferentially expressed in monocyte clusters, spanning both CD14 and CD16 monocyte compartments (Fig. 3A). CSF1R showed higher expression in CD16 monocytes, whereas LYZ, CD44, NRP1, and CD163 were more prominent in CD14 monocytes. TIMP1 and VSIG4 were expressed in both CD14 and CD16 monocyte clusters. Several mapped genes were also enriched in T-cell clusters, including CD59 and IGFBP4. In neutrophil, LCN2, PRTN3, CHI3L1, and LYZ showed relatively high expression in CD24+ARG1hi immature neutrophils, whereas CD163, CD44, CD59, and CD93 were preferentially expressed in CD163+IL1R2hi neutrophils (Fig. 3B). In tracheal aspirate scRNA-seq data, genes encoding mapped proteins were predominantly expressed in alveolar macrophages, monocyte-derived macrophages, and monocyte, with additional signals in neutrophil and epithelial compartments (Fig. 3C). Genes related to innate immunity and tissue remodeling, including VSIG4, CSF1R, CD44, FCGR3A, TIMP1, LYZ and CD163, were prominently expressed in myeloid-lineage populations, whereas several genes, such as LCN2, showed stronger expression in epithelial compartments.

## Discussion

Based on the analysis of the serum proteomic data from patients with ARDS, we identified distinct proteomic profiles associated with established ARDS subphenotypes. Beyond the known inflammatory and vascular features of hyperinflammatory ARDS, our analysis identified a serum protein pattern involving immunoregulation, tissue remodeling, complement regulation, coagulation and fibrinolysis, and metabolic alterations. Examination of external single-cell RNA-sequencing reference datasets showed that genes encoding several subphenotype-associated proteins were expressed in circulating CD14 and CD16 monocytes, distinct circulating neutrophil states, and lower-respiratory-tract monocyte/macrophage populations. These findings suggest that hyperinflammatory ARDS may be associated with a coordinated immunoregulatory protein pattern rather than a simple amplification of inflammation.

ARDS has conventionally been treated as a single disease entity. However, previous studies have demonstrated reproducible hyperinflammatory and hypoinflammatory subphenotypes defined by clinical variables and selected inflammatory, coagulation-related, endothelial, and epithelial injury biomarkers in U.S. cohorts [3–5]. Subsequent multi-omics studies have begun to clarify the biology of hyperinflammatory ARDS. Consistent with our findings, recent blood transcriptomic analyses have reported that the hyperinflammatory subphenotype is associated with innate immune activation, tissue remodeling, and metabolic dysfunction [11]. At the individual-molecule level, LCN2 was also higher in the hyperinflammatory subphenotype. A recent longitudinal blood transcriptomic and metabolic study similarly suggested that immune, metabolic, and mitochondrial pathways differ between hyperinflammatory and hypoinflammatory ARDS subphenotypes [10]. In the lower respiratory tract, hyperinflammatory ARDS has been associated with increased interferon signaling, granulopoiesis, and T-cell activation, indicating that biologic differences between subphenotypes are also present at the site of injury [22]. Notably, blood transcriptomic study has also shown that several class-defining plasma proteins did not correlate with their corresponding leukocyte transcripts [11], suggesting that circulating proteins may capture biology not fully reflected in whole-blood gene expression. An important strength of this study was the use of untargeted serum proteomics, which was not restricted to a prespecified panel of candidate proteins and enabled the simultaneous evaluation of a broad range of circulating proteins. Our findings therefore build on these reports and extend the field by providing a serum protein-level view of ARDS subphenotype biology and identifying a broader protein pattern that included several proteins associated with monocyte/macrophage biology and immunoregulation, together with complement, coagulation and fibrinolytic, and metabolic alterations beyond canonical inflammatory and vascular markers.

Among the proteins differentiating the subphenotypes, several biological programs are involved in the biology of hyperinflammatory ARDS. First, several proteins, including CSF1R, CD163, VSIG4, and TIMP1, were associated with monocyte/macrophage biology and immunoregulation. Rather than reflecting inflammation alone, these proteins suggest that the hyperinflammatory subphenotype involves immunoregulatory features. Consistent with this interpretation, a recent single-cell study of acute hypoxemic respiratory failure reported that CD163/LGMN alveolar macrophage were associated with mortality [25]. In addition, VSIG4 has been described as an immunomodulatory macrophage-associated complement receptor [26], further supporting the relevance of macrophage-linked regulatory pathways in this protein set. Genes encoding several of these proteins were also expressed in myeloid populations in the external single-cell RNA-sequencing reference datasets. However, these descriptive mappings do not identify the cellular origins of the measured serum proteins or establish differences in cell abundance or state between the ARDS subphenotypes. Consistent with these protein-level findings, pathway analysis demonstrated enrichment of MAPK signaling, cell adhesion and cytokine-response pathways, suggesting coordinated inflammatory signaling and altered intercellular communication in hyperinflammatory ARDS. In the sensitivity analysis excluding proteins correlated with IL-6 or sTNFR1, MAPK-related enrichment did not meet the significance threshold, suggesting that the MAPK signal was closely aligned with the inflammatory biomarkers used for subphenotype assignment. Second, coagulation and complement-regulatory programs were suggested by proteins such as SERPINE1 and CLU. Higher SERPINE1 abundance is consistent with impaired fibrinolytic balance, as increased PAI-1 has been associated with worse outcomes in ALI/ARDS [27, 28]. Lower CLU abundance may indicate reduced cytoprotective capacity and altered regulation of terminal complement complex formation (C5b-9/MAC). In line with this interpretation, clusterin has been shown to neutralize extracellular histones—potent mediators of cytotoxicity and thromboinflammation [29]. Third, epithelial stress, neutrophil-associated injury, and metabolic adaptation program were suggested by proteins such as LCN2 and selected metabolism-related proteins. LCN2 has been linked to acute lung inflammation and oxidative stress, and prior work supports a role for LCN2 in lung injury pathways [30]. Taken together, our findings suggest that serum proteomics extends the established view of hyperinflammatory ARDS by identifying a broader immunoregulatory protein pattern, alongside known coagulation, complement, and vascular injury pathways. This broader proteomic characterization provides a basis for future longitudinal studies to evaluate the temporal stability and clinical relevance of subphenotype-associated protein patterns.

### Limitations

This study has several potential limitations. First, the sample size was limited. Effect sizes may therefore be overestimated despite the robustness of 46 of the 54 subphenotype-associated proteins at the more stringent FDR <0.05 threshold; independent validation in a larger cohort is required. Second, we profiled serum rather than lung-compartment samples. Although circulating proteins may reflect systemic host responses and, in part, lung-derived injury signals, serum proteomics cannot directly determine the tissue or cellular origins of these proteins. To provide external cellular expression context, we examined the expression of genes encoding the subphenotype-associated serum proteins in publicly available PBMC, neutrophil, and tracheal aspirate single-cell RNA-seq datasets. These reference datasets were derived from cohorts independent of our study and provided cellular transcriptomic data rather than serum protein measurements. Therefore, transcript abundance may not correspond to circulating protein abundance, and these analyses should be considered hypothesis-generating rather than evidence of the cellular origins of the circulating proteins. Third, because subphenotype assignment and proteomic profiling were performed at a single early time point, we could not assess longitudinal transitions between subphenotypes or associated changes in proteomic patterns. Recent evidence indicates that inflammatory phenotype assignments may change during the early course of critical illness [31]. Future longitudinal studies are needed to determine how proteomic changes relate to subphenotype stability and to establish their relevance to subphenotype-guided treatment strategies. Finally, the study was conducted at a single center, which may limit generalizability. External validation in multicenter cohorts is warranted.

## Conclusion

Based on serum proteomic data from patients with ARDS, we identified distinct protein signatures associated with the hyperinflammatory subphenotype. Beyond the known inflammatory and vascular features of hyperinflammatory ARDS, the hyperinflammatory subphenotype was associated with a broader immunoregulatory protein pattern, alongside complement and fibrinolytic alterations. Examination of external single-cell RNA-sequencing reference datasets provided descriptive cellular expression context for the corresponding genes. These findings advance the serum protein-level view of ARDS subphenotype biology and provide a foundation for future longitudinal studies of the biological and clinical relevance of subphenotype-associated protein patterns.

## Supplementary Information

Additional file 1.

## Data Availability

The mass spectrometry proteomics data have been deposited in the ProteomeXchange Consortium via the PRIDE partner repository with the data set identifier PXD079948 (Available number will be determined after the acceptance of this manuscript). Reviewer access details Log in to the PRIDE website using the following details: Project accession: PXD079948 Token: elCSkAbBUjrC.

## Abbreviations

ARDS: Acute respiratory distress syndrome
APACHE: Acute Physiology and Chronic Health Evaluation
FDR: False discovery rate
GSEA: Gene set enrichment analysis
IL-6: Interleukin-6
PBMC: Peripheral blood mononuclear cell
SOFA: Sequential Organ Failure Assessment
sTNFR1: Soluble tumor necrosis factor receptor 1

## References

[1] Bos LDJ, Ware LB. Acute respiratory distress syndrome: causes, pathophysiology, and phenotypes. Lancet. 2022;400(10358):1145–56. 10.1016/S0140-6736(22)01485-4. 36070787

[2] Bellani G, Laffey JG, Pham T, et al. Epidemiology, patterns of care, and mortality for patients with acute respiratory distress syndrome in intensive care units in 50 countries. JAMA. 2016;315(8):788. 10.1001/jama.2016.0291. 26903337

[3] Calfee CS, Delucchi K, Parsons PE, Thompson BT, Ware LB, Matthay MA. Latent Class Analysis of ARDS Subphenotypes: Analysis of Data From Two Randomized Controlled Trials. Lancet Respir Med. 2014;2(8):611–20. 10.1016/S2213-2600(14)70097-9. PMC4154544 24853585

[4] Calfee CS, Delucchi KL, Sinha P, et al. ARDS Subphenotypes and Differential Response to Simvastatin: Secondary Analysis of a Randomized Controlled Trial. Lancet Respir Med. 2018;6(9):691–8. 10.1016/S2213-2600(18)30177-2. PMC6201750 30078618

[5] Famous KR, Delucchi K, Ware LB, et al. Acute respiratory distress syndrome subphenotypes respond differently to randomized fluid management strategy. Am J Respir Crit Care Med. 2017;195(3):331–8. 10.1164/rccm.201603-0645OC. PMC5328179 27513822

[6] Sinha P, Delucchi KL, Thompson BT, McAuley DF, Matthay MA. Latent class analysis of ARDS subphenotypes: a secondary analysis of the Statins for Acutely Injured Lungs from Sepsis (SAILS) study. Intensive Care Med. 2018;44(11):1859–69. 10.1007/s00134-018-5378-3. PMC6317524 30291376

[7] Sinha P, Calfee CS, Cherian S, et al. Prevalence of phenotypes of acute respiratory distress syndrome in critically ill patients with COVID-19: a prospective observational study. Lancet Respir Med. 2020;8(12):1209–18. 10.1016/S2213-2600(20)30366-0. PMC7718296 32861275

[8] Matthay MA, Zemans RL, Zimmerman GA, et al. Acute respiratory distress syndrome. Nat Rev Dis Primers. 2019;5(1):18. 10.1038/s41572-019-0069-0. PMC6709677 30872586

[9] Jabaudon M, Blondonnet R, Ware LB. Biomarkers in acute respiratory distress syndrome. Curr Opin Crit Care. 2021;27(1):46–54. 10.1097/MCC.0000000000000786. PMC7774234 33278123

[10] Alipanah-Lechner N, Neyton L, Sinha P, et al. Longitudinal multiomic signatures of ARDS and sepsis inflammatory phenotypes identify pathways associated with mortality. J Clin Invest. 2025;136(3):e196290. 10.1172/JCI196290. PMC12867137 41329523

[11] Sinha P, Neyton L, Sarma A, et al. Molecular Phenotypes of Acute Respiratory Distress Syndrome in the ROSE Trial Have Differential Outcomes and Gene Expression Patterns That Differ at Baseline and Longitudinally over Time. *Am J Respir Crit Care Med*. 209(7):816–828. 10.1164/rccm.202308-1490OC PMC10995566 38345571

[12] Vogel C, Marcotte EM. Insights into the regulation of protein abundance from proteomic and transcriptomic analyses. Nat Rev Genet. 2012;13(4):227–32. 10.1038/nrg3185. PMC3654667 22411467

[13] The ARDS Definition Task Force*. Acute Respiratory Distress Syndrome: The Berlin Definition. *JAMA*. 2012;307(23):2526–2533. 10.1001/jama.2012.5669 22797452

[14] Sinha P, Delucchi KL, McAuley DF, O’Kane CM, Matthay MA, Calfee CS. Development and Validation of Parsimonious Algorithms to Classify ARDS Phenotypes: Secondary Analyses of Randomised Controlled Trials. Lancet Respir Med. 2020;8(3):247–57. 10.1016/S2213-2600(19)30369-8. PMC7543720 31948926

[15] Yoneyama T, Ohtsuki S, Honda K, et al. Identification of IGFBP2 and IGFBP3 as compensatory biomarkers for CA19-9 in early-stage pancreatic cancer using a combination of antibody-based and LC-MS/MS-based proteomics. PLoS ONE. 2016;11(8):e0161009. 10.1371/journal.pone.0161009. PMC5007017 27579675

[16] Akai M, Maeda Y, Kawami M, Yumoto R, Takano M, Uchida Y. MiR-PAIR: microRNA-protein analysis of integrative relationship for the identification of significantly working miRNAs. Biochimica et Biophysica Acta (BBA) - General Subjects. 2025;1869(2):130746. 10.1016/j.bbagen.2024.130746. 39706375

[17] Demichev V, Messner CB, Vernardis SI, Lilley KS, Ralser M. DIA-NN: neural networks and interference correction enable deep proteome coverage in high throughput. Nat Methods. 2020;17(1):41–4. 10.1038/s41592-019-0638-x. PMC6949130 31768060

[18] Ritchie ME, Phipson B, Wu D, et al. Limma powers differential expression analyses for RNA-sequencing and microarray studies. Nucleic Acids Res. 2015;43(7):e47. 10.1093/nar/gkv007. PMC4402510 25605792

[19] Subramanian A, Tamayo P, Mootha VK, et al. Gene set enrichment analysis: a knowledge-based approach for interpreting genome-wide expression profiles. Proc Natl Acad Sci U S A. 2005;102(43):15545–50. 10.1073/pnas.0506580102. PMC1239896 16199517

[20] Jiang Y, Rosborough BR, Chen J, et al. Single cell RNA sequencing identifies an early monocyte gene signature in acute respiratory distress syndrome. JCI Insight. 2020;5(13):135678. 10.1172/jci.insight.135678. PMC7406263 32554932

[21] Sinha S, Rosin NL, Arora R, et al. Dexamethasone modulates immature neutrophils and interferon programming in severe COVID-19. Nat Med. 2022;28(1):201–11. 10.1038/s41591-021-01576-3. PMC8799469 34782790

[22] Sarma A, Christenson SA, Shoshana BZ, et al. Acute respiratory distress syndrome molecular phenotypes have distinct lower respiratory tract transcriptomes. Am J Respir Crit Care Med. 2025;211(12):2352–62. 10.1164/rccm.202407-1454OC. PMC12700240 40997225

[23] Benjamini Y, Hochberg Y. Controlling the false discovery rate: a practical and powerful approach to multiple testing. J Roy Stat Soc: Ser B (Methodol). 1995;57(1):289–300. 10.1111/j.2517-6161.1995.tb02031.x.

[24] Wildi K, Bihari S, Bersten A, et al. Early differential protein expression in blood and bronchoalveolar fluid among ARDS phenotypes. Sci Rep. 2026;16:18748. 10.1038/s41598-026-43956-0. PMC13273051 42020568

[25] Morrell ED, Holton SE, Lawrance M, et al. The transcriptional and phenotypic characteristics that define alveolar macrophage subsets in acute hypoxemic respiratory failure. Nat Commun. 2023;14:7443. 10.1038/s41467-023-43223-0. PMC10656558 37978185

[26] Reißing J, Lutz P, Frissen M, et al. Immunomodulatory receptor VSIG4 is released during spontaneous bacterial peritonitis and predicts short-term mortality. JHEP Rep. 2021;4(1):100391. 10.1016/j.jhepr.2021.100391. PMC8666561 34917912

[27] Sapru A, Curley MAQ, Brady S, Matthay MA, Flori H. Elevated PAI-1 is associated with poor clinical outcomes in pediatric patients with acute lung injury. Intensive Care Med. 2010;36(1):157–63. 10.1007/s00134-009-1690-2. PMC2807603 19855955

[28] Whyte CS, Simpson M, Morrow GB, et al. The suboptimal fibrinolytic response in COVID-19 is dictated by high PAI-1. J Thromb Haemost. 2022;20(10):2394–406. 10.1111/jth.15806. PMC9349442 35780481

[29] Augusto JF, Beauvillain C, Poli C, et al. Clusterin neutralizes the inflammatory and cytotoxic properties of extracellular histones in sepsis. Am J Respir Crit Care Med. 2023;208(2):176–87. 10.1164/rccm.202207-1253OC. 37141109

[30] Guardado S, Ojeda-Juárez D, Kaul M, Nordgren TM. Comprehensive review of lipocalin 2-mediated effects in lung inflammation. American Journal of Physiology-Lung Cellular and Molecular Physiology. 2021;321(4):L726–33. 10.1152/ajplung.00080.2021. PMC8560400 34468208

[31] van Amstel RBE, Bartek B, Vlaar APJ, et al. Temporal transitions of the hyperinflammatory and hypoinflammatory phenotypes in critical illness. Am J Respir Crit Care Med. 2025;211(3):347–56. 10.1164/rccm.202406-1241OC. PMC11936145 39642348

