# Supplementary material for: Subphenotype-specific serum proteomic signatures in patients with ARDS
Source: J Intensive Care. 2026 Oct 1;14:108. doi: 10.1186/s40560-026-00951-x (PMC13628812; doi:10.1186/s40560-026-00951-x)
Supplement: Supplementary file 1 [file 40560_2026_951_MOESM1_ESM.docx]

**Supplementary Materials**

**Subphenotype-specific serum proteomic signatures in patients with ARDS**

**Authors:** Michihito Kyo, MD, PhD; Masashi Kawami, PhD; Yasuo Uchida, PhD; Kazuya Kikutani, MD, PhD; Daisuke Kasugai, MD, PhD; Shinichiro Ohshimo, MD, PhD; Nobuyuki Hirohashi, MD, PhD; Nobuaki Shime, MD, PhD

**Table of Contents**

**Table S1.** Characteristics of single-cell RNA-seq dataset

**Table S2.** Baseline characteristics of patients with ARDS and healthy controls

**Table S3.** Descriptive comparison of serum protein abundance between patients with ARDS and healthy controls

**Table S4.** Differentially abundant proteins in hyperinflammatory ARDS compared with hypoinflammatory ARDS

**Table S5**. Sensitivity analysis of the 54 proteins differentially abundant between hyperinflammatory and hypoinflammatory ARDS subphenotypes after adjustment for APACHE II score

**Table S6**. Sensitivity of the number of differentially abundant proteins to different FDR thresholds

**Table S7**. Descriptive protein-abundance comparisons of ARDS subphenotypes with healthy controls

**Table S8**. An exploratory analysis of differentially abundant proteins between 28-day survivors and non-survivors

**Table S9.** Significantly enriched GO Biological Process pathways between ARDS subphenotypes

**Table S10.** Significantly enriched GO Biological Process pathways between ARDS subphenotypes after excluding proteins that are nominally significantly correlated with IL-6 and sTNFR1

**Figure S1.** Descriptive concordance of protein-abundance changes in the hyperinflammatory and hypoinflammatory ARDS subphenotypes relative to healthy controls

**Table S1. Characteristics of single-cell RNA-seq dataset**

| Dataset | Clinical population | Sample size | Etiology / COVID-19 status | Sampling timing | Geographic setting | Similarities to P011 | Key differences from P011 |
| --- | --- | --- | --- | --- | --- | --- | --- |
| Present cohort | Mechanically ventilated adults with Berlin-defined ARDS | 31 patients | 77% direct ARDS; 23% indirect ARDS | Within 24 h of ARDS diagnosis | Japan; single center | Reference cohort | — |
| GSE151263 | Mechanically ventilated patients with early pneumonia-associated ARDS | 3 ARDS patients used; source cohort also included 4 patients with sepsis without ARDS | Pneumonia-associated direct ARDS; pre-COVID-19 cohort | Within 24 h of intubation | United States; single center | Early sampling, invasive ventilation, and direct lung-injury context | PBMC RNA; no subphenotype classification; different institution |
| GSE200848 | Mechanically ventilated patients with non-COVID-19 ARDS | 9 patients | Non-COVID-19 ARDS | During invasive mechanical ventilation; exact timing not specified | United States; external cohort | Invasive ventilation, non-COVID-19 disease, and the same subphenotype classifier | Lower-respiratory-tract RNA; different institution |
| GSE157789 | Critically ill patients with severe bacterial or COVID-19 ARDS; present analysis restricted to early bacterial ARDS | 5 patients | Severe bacterial, non-COVID-19 ARDS | Within 72 h of ICU admission | Canada; external cohort | Early sampling, early non-COVID-19 ARDS; | Severe bacterial ARDS only; whole-blood RNA; no subphenotype classification; different institution |

**Table S2. Baseline characteristics of patients with ARDS and healthy controls**

|  | **Patients with ARDS (N=31)** | **Healthy control (N=5)** |
| --- | --- | --- |
| Age (years), median (IQR) | 67 (59–76) | 62 (61–63) |
| Male sex | 22 (71) | 2 (40) |

Data are presented as median (interquartile range) or N (%).

Abbreviations: ARDS, acute respiratory distress syndrome; IQR, interquartile range.

**Table S3. Descriptive comparison of serum protein abundance between patients with ARDS and healthy controls**

| UniProt ID | Gene symbol | Protein name | log_2_FC |
| --- | --- | --- | --- |
| P0DJI8 | SAA1 | Serum amyloid A-1 protein | 5.75 |
| P02679 | FGG | Fibrinogen gamma chain | 4.55 |
| P02675 | FGB | Fibrinogen beta chain | 4.32 |
| P02741 | CRP | C-reactive protein | 3.58 |
| P18428 | LBP | Lipopolysaccharide-binding protein | 2.87 |
| P02775 | PPBP | Platelet basic protein | -2.48 |
| P06727 | APOA4 | Apolipoprotein A-IV | -2.37 |
| P02751 | FN1 | Fibronectin | -2.32 |
| P02776 | PF4 | Platelet factor 4 | -2.31 |
| P62805 | H4C16 | Histone H4 | 2.22 |
| P35858 | IGFALS | Insulin-like growth factor-binding protein complex acid labile subunit | -2.19 |
| P02766 | TTR | Transthyretin | -2.18 |
| P04264 | KRT1 | Keratin, type II cytoskeletal 1 | 2.17 |
| P02750 | LRG1 | Leucine-rich alpha-2-glycoprotein | 2.09 |
| P05154 | SERPINA5 | Plasma serine protease inhibitor | -1.98 |
| P04196 | HRG | Histidine-rich glycoprotein | -1.97 |
| P61769 | B2M | Beta-2-microglobulin | 1.96 |
| P07988 | SFTPB | Pulmonary surfactant-associated protein B | 1.93 |
| P29622 | SERPINA4 | Kallistatin | -1.89 |
| P80108 | GPLD1 | Phosphatidylinositol-glycan-specific phospholipase D | -1.87 |
| Q86VB7 | CD163 | Scavenger receptor cysteine-rich type 1 protein M130 | 1.87 |
| P18065 | IGFBP2 | Insulin-like growth factor-binding protein 2 | 1.82 |
| Q96KN2 | CNDP1 | Beta-Ala-His dipeptidase | -1.78 |
| P19823 | ITIH2 | Inter-alpha-trypsin inhibitor heavy chain H2 | -1.77 |
| P59665 | DEFA1B | Neutrophil defensin 1 | 1.76 |
| P43652 | AFM | Afamin | -1.73 |
| Q9NQ79 | CRTAC1 | Cartilage acidic protein 1 | -1.72 |
| P02753 | RBP4 | Retinol-binding protein 4 | -1.72 |
| P01011 | SERPINA3 | Alpha-1-antichymotrypsin | 1.72 |
| Q96PD5 | PGLYRP2 | N-acetylmuramoyl-L-alanine amidase | -1.70 |
| P06276 | BCHE | Cholinesterase | -1.70 |
| Q15166 | PON3 | Serum paraoxonase/lactonase 3 | -1.69 |
| P02765 | AHSG | Alpha-2-HS-glycoprotein | -1.68 |
| P02671 | FGA | Fibrinogen alpha chain | 1.67 |
| Q02985 | CFHR3 | Complement factor H-related protein 3 | 1.67 |
| P27169 | PON1 | Serum paraoxonase/arylesterase 1 | -1.67 |
| P13796 | LCP1 | Plastin-2 | 1.66 |
| P01033 | TIMP1 | Metalloproteinase inhibitor 1 | 1.66 |
| P11597 | CETP | Cholesteryl ester transfer protein | -1.65 |
| Q9UGM5 | FETUB | Fetuin-B | -1.64 |
| P17936 | IGFBP3 | Insulin-like growth factor-binding protein 3 | -1.63 |
| Q562R1 | ACTBL2 | Beta-actin-like protein 2 | 1.63 |
| P04180 | LCAT | Phosphatidylcholine-sterol acyltransferase | -1.62 |
| P08519 | LPA | Apolipoprotein(a) | 1.61 |
| O95445 | APOM | Apolipoprotein M | -1.60 |
| P00338 | LDHA | L-lactate dehydrogenase A chain | 1.59 |
| P0DJI9 | SAA2 | Serum amyloid A-2 protein | 1.59 |
| Q04756 | HGFAC | Hepatocyte growth factor activator | -1.56 |
| P06396 | GSN | Gelsolin | -1.55 |
| P05362 | ICAM1 | Intercellular adhesion molecule 1 | 1.51 |
| P05452 | CLEC3B | Tetranectin | -1.49 |
| P40197 | GP5 | Platelet glycoprotein V | -1.45 |
| P19827 | ITIH1 | Inter-alpha-trypsin inhibitor heavy chain H1 | -1.45 |
| P00739 | HPR | Haptoglobin-related protein | -1.43 |
| P02647 | APOA1 | Apolipoprotein A-I | -1.40 |
| P04275 | VWF | von Willebrand factor | 1.40 |
| P03952 | KLKB1 | Plasma kallikrein | -1.38 |
| P05546 | SERPIND1 | Heparin cofactor 2 | -1.38 |
| P22891 | PROZ | Vitamin K-dependent protein Z | -1.37 |
| P01833 | PIGR | Polymeric immunoglobulin receptor | 1.37 |
| P02652 | APOA2 | Apolipoprotein A-II | -1.36 |
| P24821 | TNC | Tenascin | 1.34 |
| P22692 | IGFBP4 | Insulin-like growth factor-binding protein 4 | 1.33 |
| P49908 | SELENOP | Selenoprotein P | -1.32 |
| P54108 | CRISP3 | Cysteine-rich secretory protein 3 | -1.31 |
| P19320 | VCAM1 | Vascular cell adhesion protein 1 | 1.30 |
| A0A075B6I4 | IGLV10-54 | Immunoglobulin lambda variable 10-54 | -1.29 |
| O14791 | APOL1 | Apolipoprotein L1 | -1.26 |
| P07996 | THBS1 | Thrombospondin-1 | -1.26 |
| P04040 | CAT | Catalase | 1.24 |
| P05121 | SERPINE1 | Plasminogen activator inhibitor 1 | 1.24 |
| Q13103 | SPP2 | Secreted phosphoprotein 24 | -1.23 |
| P27918 | CFP | Properdin | -1.22 |
| P62937 | PPIA | Peptidyl-prolyl cis-trans isomerase A | 1.21 |
| P80188 | LCN2 | Neutrophil gelatinase-associated lipocalin | 1.21 |
| Q9UHG3 | PCYOX1 | Prenylcysteine oxidase 1 | -1.18 |
| P01034 | CST3 | Cystatin-C | 1.17 |
| P00915 | CA1 | Carbonic anhydrase 1 | 1.16 |
| P02763 | ORM1 | Alpha-1-acid glycoprotein 1 | 1.16 |
| P05160 | F13B | Coagulation factor XIII B chain | -1.15 |
| P01344 | IGF2 | Insulin-like growth factor II | -1.14 |
| P04070 | PROC | Vitamin K-dependent protein C | -1.13 |
| P07737 | PFN1 | Profilin-1 | 1.13 |
| P00748 | F12 | Coagulation factor XII | -1.11 |
| P11226 | MBL2 | Mannose-binding protein C | 1.11 |
| P02654 | APOC1 | Apolipoprotein C-I | -1.11 |
| P63104 | YWHAZ | 14-3-3 protein zeta/delta | -1.08 |
| P60985 | KRTDAP | Keratinocyte differentiation-associated protein | -1.07 |
| Q9Y5Y7 | LYVE1 | Lymphatic vessel endothelial hyaluronic acid receptor 1 | 1.05 |
| P55290 | CDH13 | Cadherin-13 | -1.04 |
| P09486 | SPARC | SPARC | -1.04 |
| P08637 | FCGR3A | Low affinity immunoglobulin gamma Fc region receptor III-A | 1.02 |
| P08709 | F7 | Coagulation factor VII | -1.00 |
| P09172 | DBH | Dopamine beta-hydroxylase | -0.99 |
| Q6UXB8 | PI16 | Peptidase inhibitor 16 | -0.99 |
| P07195 | LDHB | L-lactate dehydrogenase B chain | 0.98 |
| P35527 | KRT9 | Keratin, type I cytoskeletal 9 | 0.98 |
| P02656 | APOC3 | Apolipoprotein C-III | -0.97 |
| Q08380 | LGALS3BP | Galectin-3-binding protein | 0.97 |
| Q06033 | ITIH3 | Inter-alpha-trypsin inhibitor heavy chain H3 | 0.96 |
| P04406 | GAPDH | Glyceraldehyde-3-phosphate dehydrogenase | 0.95 |
| P00747 | PLG | Plasminogen | -0.94 |
| O75144 | ICOSLG | ICOS ligand | -0.93 |
| P02748 | C9 | Complement component C9 | 0.90 |
| P02787 | TF | Serotransferrin | -0.90 |
| P43251 | BTD | Biotinidase | -0.90 |
| O75882 | ATRN | Attractin | -0.89 |
| P04075 | ALDOA | Fructose-bisphosphate aldolase A | 0.89 |
| P00558 | PGK1 | Phosphoglycerate kinase 1 | -0.88 |
| P69891 | HBG1 | Hemoglobin subunit gamma-1 | 0.87 |
| P02749 | APOH | Beta-2-glycoprotein 1 | -0.87 |
| P08571 | CD14 | Monocyte differentiation antigen CD14 | 0.87 |
| P14625 | HSP90B1 | Endoplasmin | 0.85 |
| Q14118 | DAG1 | Dystroglycan 1 | 0.84 |
| P32119 | PRDX2 | Peroxiredoxin-2 | 0.84 |
| P33908 | MAN1A1 | Mannosyl-oligosaccharide 1,2-alpha-mannosidase IA | 0.84 |
| Q99784 | OLFM1 | Noelin | 0.82 |
| P07998 | RNASE1 | Ribonuclease pancreatic | 0.80 |
| A0A087WSX0 | IGLV5-45 | Immunoglobulin lambda variable 5-45 | -0.80 |
| P03951 | F11 | Coagulation factor XI | -0.78 |
| Q9Y6R7 | FCGBP | IgGFc-binding protein | 0.78 |
| O14498 | ISLR | Immunoglobulin superfamily containing leucine-rich repeat protein | -0.77 |
| P61981 | YWHAG | 14-3-3 protein gamma | -0.75 |
| A0A0C4DH43 | IGHV2-70D | Immunoglobulin heavy variable 2-70D | 0.75 |
| P13473 | LAMP2 | Lysosome-associated membrane glycoprotein 2 | -0.74 |
| P02768 | ALB | Albumin | -0.74 |
| P24158 | PRTN3 | Myeloblastin | 0.73 |
| P03950 | ANG | Angiogenin | -0.72 |
| A0A0A0MT96 | IGKJ3 | Immunoglobulin kappa joining 3 (Fragment) | -0.72 |
| P61626 | LYZ | Lysozyme C | 0.71 |
| O75636 | FCN3 | Ficolin-3 | -0.71 |
| P13591 | NCAM1 | Neural cell adhesion molecule 1 | -0.70 |
| P06702 | S100A9 | Protein S100-A9 | -0.70 |
| P10909 | CLU | Clusterin | -0.70 |
| P02788 | LTF | Lactotransferrin | 0.70 |
| P80723 | BASP1 | Brain acid soluble protein 1 | 0.69 |
| P01718 | IGLV3-27 | Immunoglobulin lambda variable 3-27 | -0.69 |
| Q14515 | SPARCL1 | SPARC-like protein 1 | 0.69 |
| Q16270 | IGFBP7 | Insulin-like growth factor-binding protein 7 | 0.69 |
| Q12805 | EFEMP1 | EGF-containing fibulin-like extracellular matrix protein 1 | 0.69 |
| P00918 | CA2 | Carbonic anhydrase 2 | 0.68 |
| P00734 | F2 | Prothrombin | -0.68 |
| Q9NPR2 | SEMA4B | Semaphorin-4B | 0.68 |
| Q16627 | CCL14 | C-C motif chemokine 14 | 0.67 |
| P13987 | CD59 | CD59 glycoprotein | 0.67 |
| P15291 | B4GALT1 | Beta-1,4-galactosyltransferase 1 | 0.67 |
| P01042 | KNG1 | Kininogen-1 | -0.67 |
| P05090 | APOD | Apolipoprotein D | -0.67 |
| P14618 | PKM | Pyruvate kinase PKM | 0.67 |
| P01860 | IGHG3 | Immunoglobulin heavy constant gamma 3 | -0.66 |
| A0A075B6Q5 | IGHV3-64 | Immunoglobulin heavy variable 3-64 | -0.66 |
| P01859 | IGHG2 | Immunoglobulin heavy constant gamma 2 | -0.65 |
| P55056 | APOC4 | Apolipoprotein C-IV | -0.65 |
| Q16610 | ECM1 | Extracellular matrix protein 1 | -0.65 |
| O43866 | CD5L | CD5 antigen-like | -0.65 |
| P06733 | ENO1 | Alpha-enolase | -0.64 |
| P30041 | PRDX6 | Peroxiredoxin-6 | 0.63 |
| P01880 | IGHD | Immunoglobulin heavy constant delta | -0.63 |
| P01009 | SERPINA1 | Alpha-1-antitrypsin | 0.63 |
| Q86SE8 | NPM2 | Nucleoplasmin-2 | -0.62 |
| P01742 | IGHV1-69 | Immunoglobulin heavy variable 1-69 | 0.62 |
| Q15113 | PCOLCE | Procollagen C-endopeptidase enhancer 1 | -0.61 |
| P39060 | COL18A1 | Collagen alpha-1(XVIII) chain | 0.61 |
| P07237 | P4HB | Protein disulfide-isomerase | -0.61 |
| P00488 | F13A1 | Coagulation factor XIII A chain | -0.61 |
| P24592 | IGFBP6 | Insulin-like growth factor-binding protein 6 | 0.61 |
| P00390 | GSR | Glutathione reductase, mitochondrial | 0.60 |
| Q86UD1 | OAF | Out at first protein homolog | 0.60 |
| P07339 | CTSD | Cathepsin D | 0.60 |
| A0A075B6I9 | IGLV7-46 | Immunoglobulin lambda variable 7-46 | -0.60 |
| P81605 | DCD | Dermcidin | 0.60 |
| P02655 | APOC2 | Apolipoprotein C-II | -0.59 |
| Q9NPY3 | CD93 | Complement component C1q receptor | 0.59 |
| P01766 | IGHV3-13 | Immunoglobulin heavy variable 3-13 | -0.59 |

Proteins with an absolute log_2_ fold change ≥ 0.59 were included. Positive log₂ fold changes indicate higher abundance in patients with ARDS, whereas negative values indicate higher abundance in healthy controls.

Abbreviations: FC, fold change.

**Table S4. Differentially abundant proteins in hyperinflammatory ARDS compared with hypoinflammatory ARDS**

| **UniProt_id** | **Gene symbol** | **Protein name** | **log_2_FC** | **P value** | **FDR** |
| --- | --- | --- | --- | --- | --- |
| P80188 | LCN2 | Neutrophil gelatinase-associated lipocalin | 2.01 | 7.10.E-06 | 3.10.E-03 |
| P61769 | B2M | Beta-2-microglobulin | 1.60 | 1.53.E-05 | 3.35.E-03 |
| P22692 | IGFBP4 | Insulin-like growth factor-binding protein 4 | 0.97 | 7.27.E-05 | 7.01.E-03 |
| P55058 | PLTP | Phospholipid transfer protein | 1.03 | 7.37.E-05 | 7.01.E-03 |
| P61916 | NPC2 | NPC intracellular cholesterol transporter 2 | 0.95 | 8.02.E-05 | 7.01.E-03 |
| P01033 | TIMP1 | Metalloproteinase inhibitor 1 | 1.48 | 1.12.E-04 | 8.14.E-03 |
| Q15848 | ADIPOQ | Adiponectin | 1.13 | 1.64.E-04 | 1.02.E-02 |
| P05121 | SERPINE1 | Plasminogen activator inhibitor 1 | 1.18 | 2.59.E-04 | 1.37.E-02 |
| Q12805 | EFEMP1 | EGF-containing fibulin-like extracellular matrix protein 1 | 0.86 | 3.12.E-04 | 1.37.E-02 |
| P61626 | LYZ | Lysozyme C | 1.10 | 3.22.E-04 | 1.37.E-02 |
| Q14520 | HABP2 | Hyaluronan-binding protein 2 | -0.97 | 3.44.E-04 | 1.37.E-02 |
| P16070 | CD44 | CD44 antigen | 0.75 | 4.56.E-04 | 1.57.E-02 |
| P49908 | SELENOP | Selenoprotein P | -0.79 | 4.71.E-04 | 1.57.E-02 |
| P00747 | PLG | Plasminogen | -0.97 | 5.02.E-04 | 1.57.E-02 |
| Q86VB7 | CD163 | Scavenger receptor cysteine-rich type 1 protein M130 | 1.42 | 6.51.E-04 | 1.90.E-02 |
| P04114 | APOB | Apolipoprotein B-100 | -0.90 | 7.66.E-04 | 1.90.E-02 |
| Q9Y5Y7 | LYVE1 | Lymphatic vessel endothelial hyaluronic acid receptor 1 | 1.00 | 7.67.E-04 | 1.90.E-02 |
| P02743 | APCS | Serum amyloid P-component | -0.94 | 7.81.E-04 | 1.90.E-02 |
| P41222 | PTGDS | Prostaglandin-H2 D-isomerase | 1.16 | 8.25.E-04 | 1.90.E-02 |
| P20774 | OGN | Mimecan | 0.90 | 9.39.E-04 | 2.05.E-02 |
| Q6UY14 | ADAMTSL4 | ADAMTS-like protein 4 | 0.73 | 1.03.E-03 | 2.14.E-02 |
| P03951 | F11 | Coagulation factor XI | -0.78 | 1.13.E-03 | 2.16.E-02 |
| P23470 | PTPRG | Receptor-type tyrosine-protein phosphatase gamma | 0.60 | 1.16.E-03 | 2.16.E-02 |
| P36222 | CHI3L1 | Chitinase-3-like protein 1 | 1.24 | 1.19.E-03 | 2.16.E-02 |
| Q9NPY3 | CD93 | Complement component C1q receptor | 0.80 | 1.36.E-03 | 2.35.E-02 |
| Q9Y279 | VSIG4 | V-set and immunoglobulin domain-containing protein 4 | 1.25 | 1.40.E-03 | 2.35.E-02 |
| Q16270 | IGFBP7 | Insulin-like growth factor-binding protein 7 | 0.84 | 1.59.E-03 | 2.47.E-02 |
| P07998 | RNASE1 | Ribonuclease pancreatic | 0.99 | 1.59.E-03 | 2.47.E-02 |
| P15291 | B4GALT1 | Beta-1,4-galactosyltransferase 1 | 0.82 | 1.64.E-03 | 2.47.E-02 |
| P10909 | CLU | Clusterin | -0.73 | 1.75.E-03 | 2.56.E-02 |
| P13671 | C6 | Complement component C6 | -0.79 | 1.82.E-03 | 2.57.E-02 |
| P02748 | C9 | Complement component C9 | -0.90 | 2.05.E-03 | 2.76.E-02 |
| P05546 | SERPIND1 | Heparin cofactor 2 | -1.09 | 2.09.E-03 | 2.76.E-02 |
| P12318 | FCGR2A | Low affinity immunoglobulin gamma Fc region receptor II-a | 0.73 | 2.47.E-03 | 3.06.E-02 |
| P24158 | PRTN3 | Myeloblastin | 1.27 | 2.53.E-03 | 3.06.E-02 |
| P01034 | CST3 | Cystatin-C | 0.91 | 2.59.E-03 | 3.06.E-02 |
| P60985 | KRTDAP | Keratinocyte differentiation-associated protein | 0.63 | 2.74.E-03 | 3.15.E-02 |
| P02649 | APOE | Apolipoprotein E | -0.95 | 2.87.E-03 | 3.18.E-02 |
| P43121 | MCAM | Cell surface glycoprotein MUC18 | 0.63 | 2.95.E-03 | 3.18.E-02 |
| O14786 | NRP1 | Neuropilin-1 | 0.70 | 2.98.E-03 | 3.18.E-02 |
| P55056 | APOC4 | Apolipoprotein C-IV | -1.20 | 3.16.E-03 | 3.29.E-02 |
| P12259 | F5 | Coagulation factor V | -0.76 | 3.28.E-03 | 3.33.E-02 |
| P00488 | F13A1 | Coagulation factor XIII A chain | 1.10 | 3.84.E-03 | 3.82.E-02 |
| P00740 | F9 | Coagulation factor IX | -0.76 | 4.12.E-03 | 4.01.E-02 |
| P07333 | CSF1R | Macrophage colony-stimulating factor 1 receptor | 0.79 | 5.06.E-03 | 4.67.E-02 |
| P13987 | CD59 | CD59 glycoprotein | 0.89 | 5.13.E-03 | 4.67.E-02 |
| Q15063 | POSTN | Periostin | 0.80 | 6.98.E-03 | 6.23.E-02 |
| Q9UHG3 | PCYOX1 | Prenylcysteine oxidase 1 | -0.75 | 9.38.E-03 | 7.88.E-02 |
| P07737 | PFN1 | Profilin-1 | 0.91 | 1.06.E-02 | 8.71.E-02 |
| P08637 | FCGR3A | Low affinity immunoglobulin gamma Fc region receptor III-A | 0.75 | 1.14.E-02 | 9.05.E-02 |
| P19320 | VCAM1 | Vascular cell adhesion protein 1 | 0.81 | 1.14.E-02 | 9.05.E-02 |
| P19827 | ITIH1 | Inter-alpha-trypsin inhibitor heavy chain H1 | -0.80 | 1.26.E-02 | 9.83.E-02 |
| Q15828 | CST6 | Cystatin-M | 0.69 | 1.30.E-02 | 9.97.E-02 |
| P19823 | ITIH2 | Inter-alpha-trypsin inhibitor heavy chain H2 | -0.99 | 1.33.E-02 | 1.00.E-01 |

Proteins with FDR < 0.10 and an absolute log_2_ fold change ≥ 0.59 were included.

Abbreviations: FC, fold change; FDR, false discovery rate.

**Table S5. Sensitivity analysis of the 54 proteins differentially abundant between hyperinflammatory and hypoinflammatory ARDS subphenotypes after adjustment for APACHE II score**

| **UniProt_id** | **Gene symbol** | **Protein name** | **log_2_FC** | **P value** | **FDR** |
| --- | --- | --- | --- | --- | --- |
| P55058 | PLTP | Phospholipid transfer protein | 1.14 | 3.17E-04 | 4.90E-02 |
| P02743 | APCS | Serum amyloid P-component | -1.17 | 6.63E-04 | 4.90E-02 |
| P10909 | CLU | Clusterin | -0.98 | 6.79E-04 | 4.90E-02 |
| Q9Y5Y7 | LYVE1 | Lymphatic vessel endothelial hyaluronic acid receptor 1 | 1.23 | 7.62E-04 | 4.90E-02 |
| P04114 | APOB | Apolipoprotein B-100 | -1.10 | 8.71E-04 | 4.90E-02 |
| P03951 | F11 | Coagulation factor XI | -0.97 | 9.04E-04 | 4.90E-02 |
| P80188 | LCN2 | Neutrophil gelatinase-associated lipocalin | 1.70 | 9.19E-04 | 4.90E-02 |
| P07333 | CSF1R | Macrophage colony-stimulating factor 1 receptor | 1.12 | 1.06E-03 | 4.90E-02 |
| P00747 | PLG | Plasminogen | -1.12 | 1.15E-03 | 4.90E-02 |
| Q9NPY3 | CD93 | Complement component C1q receptor | 0.99 | 1.27E-03 | 4.90E-02 |
| Q86VB7 | CD163 | Scavenger receptor cysteine-rich type 1 protein M130 | 1.64 | 1.35E-03 | 4.90E-02 |
| P19823 | ITIH2 | Inter-alpha-trypsin inhibitor heavy chain H2 | -1.53 | 1.62E-03 | 5.39E-02 |
| P61769 | B2M | Beta-2-microglobulin | 1.33 | 1.73E-03 | 5.39E-02 |
| P12259 | F5 | Coagulation factor V | -0.99 | 1.89E-03 | 5.50E-02 |
| Q12805 | EFEMP1 | EGF-containing fibulin-like extracellular matrix protein 1 | 0.89 | 2.19E-03 | 5.71E-02 |
| Q15848 | ADIPOQ | Adiponectin | 1.11 | 2.31E-03 | 5.71E-02 |
| P19827 | ITIH1 | Inter-alpha-trypsin inhibitor heavy chain H1 | -1.20 | 2.35E-03 | 5.71E-02 |
| P05546 | SERPIND1 | Heparin cofactor 2 | -1.32 | 2.59E-03 | 5.96E-02 |
| P15291 | B4GALT1 | Beta-1,4-galactosyltransferase 1 | 0.94 | 3.17E-03 | 6.71E-02 |
| Q9UHG3 | PCYOX1 | Prenylcysteine oxidase 1 | -1.05 | 3.22E-03 | 6.71E-02 |
| P16070 | CD44 | CD44 antigen | 0.75 | 4.03E-03 | 7.62E-02 |
| P49908 | SELENOP | Selenoprotein P | -0.79 | 4.05E-03 | 7.62E-02 |
| P43121 | MCAM | Cell surface glycoprotein MUC18 | 0.74 | 4.51E-03 | 7.62E-02 |
| P01033 | TIMP1 | Metalloproteinase inhibitor 1 | 1.27 | 4.75E-03 | 7.62E-02 |
| O14786 | NRP1 | Neuropilin-1 | 0.81 | 4.96E-03 | 7.62E-02 |
| P00740 | F9 | Coagulation factor IX | -0.91 | 5.21E-03 | 7.62E-02 |
| P02649 | APOE | Apolipoprotein E | -1.09 | 5.36E-03 | 7.62E-02 |
| P61626 | LYZ | Lysozyme C | 0.98 | 7.14E-03 | 8.92E-02 |
| P22692 | IGFBP4 | Insulin-like growth factor-binding protein 4 | 0.75 | 7.39E-03 | 8.97E-02 |
| P12318 | FCGR2A | Low affinity immunoglobulin gamma Fc region receptor II-a | 0.78 | 8.63E-03 | 9.67E-02 |
| P19320 | VCAM1 | Vascular cell adhesion protein 1 | 1.02 | 1.00E-02 | 1.03E-01 |
| P05121 | SERPINE1 | Plasminogen activator inhibitor 1 | 0.97 | 1.03E-02 | 1.03E-01 |
| P55056 | APOC4 | Apolipoprotein C-IV | -1.28 | 1.04E-02 | 1.03E-01 |
| Q14520 | HABP2 | Hyaluronan-binding protein 2 | -0.82 | 1.05E-02 | 1.03E-01 |
| Q6UY14 | ADAMTSL4 | ADAMTS-like protein 4 | 0.68 | 1.14E-02 | 1.03E-01 |
| P02748 | C9 | Complement component C9 | -0.90 | 1.16E-02 | 1.03E-01 |
| P60985 | KRTDAP | Keratinocyte differentiation-associated protein | 0.64 | 1.29E-02 | 1.08E-01 |
| P13671 | C6 | Complement component C6 | -0.77 | 1.35E-02 | 1.10E-01 |
| P00488 | F13A1 | Coagulation factor XIII A chain | 1.15 | 1.45E-02 | 1.15E-01 |
| P23470 | PTPRG | Receptor-type tyrosine-protein phosphatase gamma | 0.52 | 1.88E-02 | 1.45E-01 |
| P08637 | FCGR3A | Low affinity immunoglobulin gamma Fc region receptor III-A | 0.86 | 1.96E-02 | 1.45E-01 |
| P07998 | RNASE1 | Ribonuclease pancreatic | 0.86 | 2.23E-02 | 1.57E-01 |
| Q15063 | POSTN | Periostin | 0.81 | 2.71E-02 | 1.79E-01 |
| P24158 | PRTN3 | Myeloblastin | 1.06 | 3.58E-02 | 2.07E-01 |
| P36222 | CHI3L1 | Chitinase-3-like protein 1 | 0.90 | 4.31E-02 | 2.25E-01 |
| P41222 | PTGDS | Prostaglandin-H2 D-isomerase | 0.79 | 4.71E-02 | 2.39E-01 |
| P20774 | OGN | Mimecan | 0.60 | 5.26E-02 | 2.61E-01 |
| Q9Y279 | VSIG4 | V-set and immunoglobulin domain-containing protein 4 | 0.88 | 5.37E-02 | 2.61E-01 |
| Q16270 | IGFBP7 | Insulin-like growth factor-binding protein 7 | 0.56 | 6.54E-02 | 2.74E-01 |
| P61916 | NPC2 | NPC intracellular cholesterol transporter 2 | 0.40 | 6.71E-02 | 2.77E-01 |
| P01034 | CST3 | Cystatin-C | 0.63 | 7.48E-02 | 2.80E-01 |
| P07737 | PFN1 | Profilin-1 | 0.73 | 8.77E-02 | 3.14E-01 |
| Q15828 | CST6 | Cystatin-M | 0.48 | 1.53E-01 | 4.03E-01 |
| P13987 | CD59 | CD59 glycoprotein | 0.47 | 1.92E-01 | 4.44E-01 |

Abbreviations: FC, fold change; FDR, false discovery rate.

**Table S6. Sensitivity of the number of differentially abundant proteins to different FDR thresholds**

| FDR threshold | Number of differentially abundant proteins |
| --- | --- |
| FDR < 0.01 | 6 |
| FDR < 0.05 | 46 |
| FDR < 0.10 | 54 |

Abbreviations: FDR, false discovery rate.

**Table S7. Descriptive protein-abundance comparisons of ARDS subphenotypes with healthy controls**

| UniProt_id | Gene symbol | Protein name | log_2_FC  (Hyper vs healthy control) | log_2_FC  (Hypo vs healthy control) | log_2_FC  (ARDS vs healthy control) | Direction concordance |
| --- | --- | --- | --- | --- | --- | --- |
| P0DJI8 | SAA1 | Serum amyloid A-1 protein | 5.39 | 6.01 | 5.75 | Concordant across all three comparisons |
| P02679 | FGG | Fibrinogen gamma chain | 5.50 | 3.87 | 4.55 | Concordant across all three comparisons |
| P02675 | FGB | Fibrinogen beta chain | 5.28 | 3.63 | 4.32 | Concordant across all three comparisons |
| P02741 | CRP | C-reactive protein | 3.94 | 3.32 | 3.58 | Concordant across all three comparisons |
| P18428 | LBP | Lipopolysaccharide-binding protein | 2.99 | 2.79 | 2.87 | Concordant across all three comparisons |
| P02775 | PPBP | Platelet basic protein | -2.75 | -2.29 | -2.48 | Concordant across all three comparisons |
| P06727 | APOA4 | Apolipoprotein A-IV | -1.96 | -2.67 | -2.37 | Concordant across all three comparisons |
| P02751 | FN1 | Fibronectin | -2.97 | -1.86 | -2.32 | Concordant across all three comparisons |
| P02776 | PF4 | Platelet factor 4 | -2.69 | -2.04 | -2.31 | Concordant across all three comparisons |
| P62805 | H4C16 | Histone H4 | 2.30 | 2.17 | 2.22 | Concordant across all three comparisons |
| P35858 | IGFALS | Insulin-like growth factor-binding protein complex acid labile subunit | -2.01 | -2.32 | -2.19 | Concordant across all three comparisons |
| P02766 | TTR | Transthyretin | -2.17 | -2.19 | -2.18 | Concordant across all three comparisons |
| P04264 | KRT1 | Keratin, type II cytoskeletal 1 | 1.91 | 2.36 | 2.17 | Concordant across all three comparisons |
| P02750 | LRG1 | Leucine-rich alpha-2-glycoprotein | 2.11 | 2.09 | 2.09 | Concordant across all three comparisons |
| P05154 | SERPINA5 | Plasma serine protease inhibitor | -2.39 | -1.68 | -1.98 | Concordant across all three comparisons |
| P04196 | HRG | Histidine-rich glycoprotein | -2.11 | -1.87 | -1.97 | Concordant across all three comparisons |
| P61769 | B2M | Beta-2-microglobulin | 2.88 | 1.29 | 1.96 | Concordant across all three comparisons |
| P07988 | SFTPB | Pulmonary surfactant-associated protein B | 1.75 | 2.06 | 1.93 | Concordant across all three comparisons |
| P29622 | SERPINA4 | Kallistatin | -2.25 | -1.64 | -1.89 | Concordant across all three comparisons |
| P80108 | GPLD1 | Phosphatidylinositol-glycan-specific phospholipase D | -2.04 | -1.75 | -1.87 | Concordant across all three comparisons |
| Q86VB7 | CD163 | Scavenger receptor cysteine-rich type 1 protein M130 | 2.69 | 1.28 | 1.87 | Concordant across all three comparisons |
| P18065 | IGFBP2 | Insulin-like growth factor-binding protein 2 | 2.18 | 1.56 | 1.82 | Concordant across all three comparisons |
| Q96KN2 | CNDP1 | Beta-Ala-His dipeptidase | -2.20 | -1.48 | -1.78 | Concordant across all three comparisons |
| P19823 | ITIH2 | Inter-alpha-trypsin inhibitor heavy chain H2 | -2.34 | -1.35 | -1.77 | Concordant across all three comparisons |
| P59665 | DEFA1B | Neutrophil defensin 1 | 2.26 | 1.40 | 1.76 | Concordant across all three comparisons |
| P43652 | AFM | Afamin | -1.86 | -1.64 | -1.74 | Concordant across all three comparisons |
| Q9NQ79 | CRTAC1 | Cartilage acidic protein 1 | -1.61 | -1.81 | -1.72 | Concordant across all three comparisons |
| P02753 | RBP4 | Retinol-binding protein 4 | -1.86 | -1.62 | -1.72 | Concordant across all three comparisons |
| P01011 | SERPINA3 | Alpha-1-antichymotrypsin | 1.55 | 1.83 | 1.72 | Concordant across all three comparisons |
| Q96PD5 | PGLYRP2 | N-acetylmuramoyl-L-alanine amidase | -1.96 | -1.52 | -1.71 | Concordant across all three comparisons |
| P06276 | BCHE | Cholinesterase | -1.84 | -1.59 | -1.70 | Concordant across all three comparisons |
| Q15166 | PON3 | Serum paraoxonase/lactonase 3 | -2.06 | -1.42 | -1.69 | Concordant across all three comparisons |
| P02765 | AHSG | Alpha-2-HS-glycoprotein | -1.80 | -1.58 | -1.68 | Concordant across all three comparisons |
| P02671 | FGA | Fibrinogen alpha chain | 2.39 | 1.15 | 1.67 | Concordant across all three comparisons |
| Q02985 | CFHR3 | Complement factor H-related protein 3 | 1.82 | 1.56 | 1.67 | Concordant across all three comparisons |
| P27169 | PON1 | Serum paraoxonase/arylesterase 1 | -2.03 | -1.41 | -1.67 | Concordant across all three comparisons |
| P13796 | LCP1 | Plastin-2 | 1.83 | 1.54 | 1.66 | Concordant across all three comparisons |
| P01033 | TIMP1 | Metalloproteinase inhibitor 1 | 2.51 | 1.04 | 1.66 | Concordant across all three comparisons |
| P11597 | CETP | Cholesteryl ester transfer protein | -1.54 | -1.73 | -1.65 | Concordant across all three comparisons |
| Q9UGM5 | FETUB | Fetuin-B | -2.02 | -1.37 | -1.64 | Concordant across all three comparisons |
| P17936 | IGFBP3 | Insulin-like growth factor-binding protein 3 | -1.49 | -1.73 | -1.63 | Concordant across all three comparisons |
| Q562R1 | ACTBL2 | Beta-actin-like protein 2 | 1.90 | 1.44 | 1.63 | Concordant across all three comparisons |
| P04180 | LCAT | Phosphatidylcholine-sterol acyltransferase | -2.00 | -1.36 | -1.62 | Concordant across all three comparisons |
| P08519 | LPA | Apolipoprotein(a) | 1.45 | 1.72 | 1.61 | Concordant across all three comparisons |
| O95445 | APOM | Apolipoprotein M | -1.81 | -1.45 | -1.60 | Concordant across all three comparisons |
| P00338 | LDHA | L-lactate dehydrogenase A chain | 1.69 | 1.52 | 1.59 | Concordant across all three comparisons |
| P0DJI9 | SAA2 | Serum amyloid A-2 protein | 1.34 | 1.77 | 1.59 | Concordant across all three comparisons |
| Q04756 | HGFAC | Hepatocyte growth factor activator | -1.77 | -1.41 | -1.56 | Concordant across all three comparisons |
| P06396 | GSN | Gelsolin | -1.47 | -1.61 | -1.55 | Concordant across all three comparisons |
| P05362 | ICAM1 | Intercellular adhesion molecule 1 | 1.64 | 1.41 | 1.51 | Concordant across all three comparisons |
| P05452 | CLEC3B | Tetranectin | -1.44 | -1.53 | -1.49 | Concordant across all three comparisons |
| P40197 | GP5 | Platelet glycoprotein V | -1.50 | -1.41 | -1.45 | Concordant across all three comparisons |
| P19827 | ITIH1 | Inter-alpha-trypsin inhibitor heavy chain H1 | -1.92 | -1.11 | -1.45 | Concordant across all three comparisons |
| P00739 | HPR | Haptoglobin-related protein | -1.29 | -1.53 | -1.43 | Concordant across all three comparisons |
| P02647 | APOA1 | Apolipoprotein A-I | -1.56 | -1.29 | -1.40 | Concordant across all three comparisons |
| P04275 | VWF | von Willebrand factor | 1.43 | 1.38 | 1.40 | Concordant across all three comparisons |
| P03952 | KLKB1 | Plasma kallikrein | -1.71 | -1.14 | -1.38 | Concordant across all three comparisons |
| P05546 | SERPIND1 | Heparin cofactor 2 | -2.01 | -0.92 | -1.38 | Concordant across all three comparisons |
| P22891 | PROZ | Vitamin K-dependent protein Z | -1.80 | -1.06 | -1.37 | Concordant across all three comparisons |
| P01833 | PIGR | Polymeric immunoglobulin receptor | 1.18 | 1.51 | 1.37 | Concordant across all three comparisons |
| P02652 | APOA2 | Apolipoprotein A-II | -1.38 | -1.34 | -1.36 | Concordant across all three comparisons |
| P24821 | TNC | Tenascin | 1.69 | 1.09 | 1.34 | Concordant across all three comparisons |
| P22692 | IGFBP4 | Insulin-like growth factor-binding protein 4 | 1.89 | 0.92 | 1.33 | Concordant across all three comparisons |
| P49908 | SELENOP | Selenoprotein P | -1.77 | -0.99 | -1.32 | Concordant across all three comparisons |
| P54108 | CRISP3 | Cysteine-rich secretory protein 3 | -1.03 | -1.51 | -1.31 | Concordant across all three comparisons |
| P19320 | VCAM1 | Vascular cell adhesion protein 1 | 1.77 | 0.96 | 1.30 | Concordant across all three comparisons |
| A0A075B6I4 | IGLV10-54 | Immunoglobulin lambda variable 10-54 | -1.67 | -1.02 | -1.29 | Concordant across all three comparisons |
| O14791 | APOL1 | Apolipoprotein L1 | -1.41 | -1.16 | -1.26 | Concordant across all three comparisons |
| P07996 | THBS1 | Thrombospondin-1 | -1.51 | -1.08 | -1.26 | Concordant across all three comparisons |
| P04040 | CAT | Catalase | 1.14 | 1.32 | 1.24 | Concordant across all three comparisons |
| P05121 | SERPINE1 | Plasminogen activator inhibitor 1 | 1.92 | 0.74 | 1.24 | Concordant across all three comparisons |
| Q13103 | SPP2 | Secreted phosphoprotein 24 | -1.53 | -1.02 | -1.23 | Concordant across all three comparisons |
| P27918 | CFP | Properdin | -1.13 | -1.29 | -1.22 | Concordant across all three comparisons |
| P62937 | PPIA | Peptidyl-prolyl cis-trans isomerase A | 1.62 | 0.92 | 1.21 | Concordant across all three comparisons |
| Q9UHG3 | PCYOX1 | Prenylcysteine oxidase 1 | -1.62 | -0.86 | -1.18 | Concordant across all three comparisons |
| P01034 | CST3 | Cystatin-C | 1.70 | 0.79 | 1.17 | Concordant across all three comparisons |
| P00915 | CA1 | Carbonic anhydrase 1 | 0.82 | 1.41 | 1.16 | Concordant across all three comparisons |
| P02763 | ORM1 | Alpha-1-acid glycoprotein 1 | 1.04 | 1.24 | 1.16 | Concordant across all three comparisons |
| P05160 | F13B | Coagulation factor XIII B chain | -1.17 | -1.13 | -1.15 | Concordant across all three comparisons |
| P01344 | IGF2 | Insulin-like growth factor II | -1.18 | -1.11 | -1.14 | Concordant across all three comparisons |
| P04070 | PROC | Vitamin K-dependent protein C | -1.42 | -0.92 | -1.13 | Concordant across all three comparisons |
| P07737 | PFN1 | Profilin-1 | 1.66 | 0.75 | 1.13 | Concordant across all three comparisons |
| P00748 | F12 | Coagulation factor XII | -1.17 | -1.06 | -1.11 | Concordant across all three comparisons |
| P11226 | MBL2 | Mannose-binding protein C | 0.91 | 1.25 | 1.11 | Concordant across all three comparisons |
| P02654 | APOC1 | Apolipoprotein C-I | -1.48 | -0.83 | -1.11 | Concordant across all three comparisons |
| P63104 | YWHAZ | 14-3-3 protein zeta/delta | -1.04 | -1.11 | -1.08 | Concordant across all three comparisons |
| P60985 | KRTDAP | Keratinocyte differentiation-associated protein | -0.71 | -1.34 | -1.07 | Concordant across all three comparisons |
| Q9Y5Y7 | LYVE1 | Lymphatic vessel endothelial hyaluronic acid receptor 1 | 1.63 | 0.64 | 1.05 | Concordant across all three comparisons |
| P55290 | CDH13 | Cadherin-13 | -0.77 | -1.24 | -1.04 | Concordant across all three comparisons |
| P09486 | SPARC | SPARC | -1.10 | -1.00 | -1.04 | Concordant across all three comparisons |
| P08637 | FCGR3A | Low affinity immunoglobulin gamma Fc region receptor III-A | 1.46 | 0.70 | 1.02 | Concordant across all three comparisons |
| P08709 | F7 | Coagulation factor VII | -1.17 | -0.87 | -1.00 | Concordant across all three comparisons |
| P09172 | DBH | Dopamine beta-hydroxylase | -1.09 | -0.93 | -0.99 | Concordant across all three comparisons |
| Q6UXB8 | PI16 | Peptidase inhibitor 16 | -0.62 | -1.25 | -0.99 | Concordant across all three comparisons |
| P07195 | LDHB | L-lactate dehydrogenase B chain | 0.95 | 1.00 | 0.98 | Concordant across all three comparisons |
| P35527 | KRT9 | Keratin, type I cytoskeletal 9 | 0.83 | 1.09 | 0.98 | Concordant across all three comparisons |
| P02656 | APOC3 | Apolipoprotein C-III | -1.46 | -0.62 | -0.97 | Concordant across all three comparisons |
| Q08380 | LGALS3BP | Galectin-3-binding protein | 0.72 | 1.14 | 0.97 | Concordant across all three comparisons |
| Q06033 | ITIH3 | Inter-alpha-trypsin inhibitor heavy chain H3 | 0.74 | 1.12 | 0.96 | Concordant across all three comparisons |
| P04406 | GAPDH | Glyceraldehyde-3-phosphate dehydrogenase | 0.86 | 1.01 | 0.95 | Concordant across all three comparisons |
| O75144 | ICOSLG | ICOS ligand | -0.63 | -1.15 | -0.93 | Concordant across all three comparisons |
| P02787 | TF | Serotransferrin | -1.03 | -0.81 | -0.90 | Concordant across all three comparisons |
| P43251 | BTD | Biotinidase | -1.03 | -0.80 | -0.90 | Concordant across all three comparisons |
| O75882 | ATRN | Attractin | -0.83 | -0.93 | -0.89 | Concordant across all three comparisons |
| P04075 | ALDOA | Fructose-bisphosphate aldolase A | 1.19 | 0.67 | 0.89 | Concordant across all three comparisons |
| P69891 | HBG1 | Hemoglobin subunit gamma-1 | 0.85 | 0.89 | 0.87 | Concordant across all three comparisons |
| P08571 | CD14 | Monocyte differentiation antigen CD14 | 1.06 | 0.73 | 0.87 | Concordant across all three comparisons |
| P14625 | HSP90B1 | Endoplasmin | 0.98 | 0.75 | 0.85 | Concordant across all three comparisons |
| P33908 | MAN1A1 | Mannosyl-oligosaccharide 1,2-alpha-mannosidase IA | 0.77 | 0.88 | 0.84 | Concordant across all three comparisons |
| Q99784 | OLFM1 | Noelin | 0.95 | 0.72 | 0.82 | Concordant across all three comparisons |
| O14498 | ISLR | Immunoglobulin superfamily containing leucine-rich repeat protein | -0.64 | -0.87 | -0.77 | Concordant across all three comparisons |
| P61981 | YWHAG | 14-3-3 protein gamma | -0.80 | -0.72 | -0.76 | Concordant across all three comparisons |
| A0A0C4DH43 | IGHV2-70D | Immunoglobulin heavy variable 2-70D | 0.75 | 0.75 | 0.75 | Concordant across all three comparisons |
| P13473 | LAMP2 | Lysosome-associated membrane glycoprotein 2 | -0.62 | -0.83 | -0.74 | Concordant across all three comparisons |
| P02768 | ALB | Albumin | -0.64 | -0.81 | -0.74 | Concordant across all three comparisons |
| P03950 | ANG | Angiogenin | -0.84 | -0.64 | -0.72 | Concordant across all three comparisons |
| O75636 | FCN3 | Ficolin-3 | -0.72 | -0.70 | -0.71 | Concordant across all three comparisons |
| P06702 | S100A9 | Protein S100-A9 | -0.66 | -0.73 | -0.70 | Concordant across all three comparisons |
| P01718 | IGLV3-27 | Immunoglobulin lambda variable 3-27 | -0.63 | -0.74 | -0.69 | Concordant across all three comparisons |
| P00918 | CA2 | Carbonic anhydrase 2 | 0.75 | 0.64 | 0.69 | Concordant across all three comparisons |
| A0A075B6Q5 | IGHV3-64 | Immunoglobulin heavy variable 3-64 | -0.75 | -0.59 | -0.66 | Concordant across all three comparisons |

Proteins showing an absolute log2 fold change >0.59 across ARDS subphenotype–healthy control comparisons

Abbreviations: FC, fold change.

**Table S8. An exploratory analysis of differentially abundant proteins between 28-day survivors and non-survivors**

| UniProt_id | Gene symbol | Protein name | log_2_FC  (non-survivors vs survivors) | P value | FDR | Overlap with 54-protein subphenotype signature | log_2_FC  (ARDS vs healthy control) | Direction concordant (mortality vs ARDS-control) |
| --- | --- | --- | --- | --- | --- | --- | --- | --- |
| P17936 | IGFBP3 | Insulin-like growth factor-binding protein 3 | -1.02 | 0.0005 | 0.095 | No | -1.63 | Yes |
| P22891 | PROZ | Vitamin K-dependent protein Z | -1.20 | 0.0006 | 0.095 | No | -1.37 | Yes |
| Q92820 | GGH | Gamma-glutamyl hydrolase | -0.84 | 0.0010 | 0.095 | No | -0.23 | Yes |
| P01344 | IGF2 | Insulin-like growth factor II | -0.94 | 0.0010 | 0.095 | No | -1.14 | Yes |
| P35858 | IGFALS | Insulin-like growth factor-binding protein complex acid labile subunit | -1.44 | 0.0014 | 0.095 | No | -2.19 | Yes |
| P05121 | SERPINE1 | Plasminogen activator inhibitor 1 | 1.09 | 0.0015 | 0.095 | Yes | 1.24 | Yes |
| Q96KN2 | CNDP1 | Beta-Ala-His dipeptidase | -1.36 | 0.0016 | 0.095 | No | -1.78 | Yes |
| Q99969 | RARRES2 | Retinoic acid receptor responder protein 2 | -0.96 | 0.0017 | 0.095 | No | 0.04 | No |

Abbreviations: FC, fold change; FDR, false discovery rate.

**Table S9. Significantly enriched GO Biological Process pathways between ARDS subphenotypes**

| **ID** | **Description** | **Set size** | **Enrichment score** | **NES** | **FDR** |
| --- | --- | --- | --- | --- | --- |
| GO:0043410 | positive regulation of MAPK cascade | 21 | 0.71 | 2.12 | 0.01 |
| GO:0043408 | regulation of MAPK cascade | 28 | 0.66 | 2.08 | 0.01 |
| GO:0000165 | MAPK cascade | 30 | 0.64 | 2.06 | 0.01 |
| GO:0098609 | cell-cell adhesion | 47 | 0.58 | 2.03 | 0.01 |
| GO:0007155 | cell adhesion | 88 | 0.51 | 1.98 | 0.01 |
| GO:0008219 | cell death | 59 | 0.51 | 1.87 | 0.01 |
| GO:0012501 | programmed cell death | 59 | 0.51 | 1.87 | 0.01 |
| GO:0023052 | signaling | 147 | 0.43 | 1.76 | 0.01 |
| GO:0007165 | signal transduction | 141 | 0.41 | 1.69 | 0.01 |
| GO:0050794 | regulation of cellular process | 221 | 0.40 | 1.68 | 0.01 |
| GO:0042127 | regulation of cell population proliferation | 72 | 0.49 | 1.83 | 0.01 |
| GO:0008283 | cell population proliferation | 92 | 0.46 | 1.79 | 0.01 |
| GO:0007154 | cell communication | 151 | 0.42 | 1.73 | 0.01 |
| GO:0048523 | negative regulation of cellular process | 127 | 0.42 | 1.71 | 0.01 |
| GO:0048522 | positive regulation of cellular process | 144 | 0.40 | 1.66 | 0.01 |
| GO:0009628 | response to abiotic stimulus | 34 | 0.62 | 2.05 | 0.01 |
| GO:0022409 | positive regulation of cell-cell adhesion | 17 | 0.72 | 2.03 | 0.01 |
| GO:0002455 | humoral immune response mediated by circulating immunoglobulin | 32 | -0.54 | -2.00 | 0.01 |
| GO:0009888 | tissue development | 66 | 0.49 | 1.83 | 0.02 |
| GO:0046486 | glycerolipid metabolic process | 20 | -0.61 | -1.99 | 0.02 |
| GO:0070374 | positive regulation of ERK1 and ERK2 cascade | 13 | 0.76 | 1.97 | 0.02 |
| GO:0007267 | cell-cell signaling | 32 | 0.60 | 1.95 | 0.02 |
| GO:0006958 | complement activation, classical pathway | 31 | -0.52 | -1.93 | 0.02 |
| GO:0030155 | regulation of cell adhesion | 48 | 0.54 | 1.92 | 0.02 |
| GO:0022407 | regulation of cell-cell adhesion | 24 | 0.63 | 1.91 | 0.02 |
| GO:0010646 | regulation of cell communication | 85 | 0.45 | 1.72 | 0.02 |
| GO:0023051 | regulation of signaling | 85 | 0.45 | 1.72 | 0.02 |
| GO:0006915 | apoptotic process | 55 | 0.51 | 1.84 | 0.02 |
| GO:0034097 | response to cytokine | 33 | 0.58 | 1.90 | 0.02 |
| GO:0035556 | intracellular signal transduction | 54 | 0.51 | 1.82 | 0.02 |
| GO:0071345 | cellular response to cytokine stimulus | 29 | 0.60 | 1.90 | 0.02 |
| GO:0010033 | response to organic substance | 102 | 0.43 | 1.68 | 0.02 |
| GO:0071310 | cellular response to organic substance | 59 | 0.49 | 1.79 | 0.02 |
| GO:0044248 | cellular catabolic process | 47 | -0.43 | -1.76 | 0.02 |
| GO:0070663 | regulation of leukocyte proliferation | 11 | 0.78 | 1.95 | 0.02 |
| GO:0006641 | triglyceride metabolic process | 14 | -0.66 | -1.96 | 0.03 |
| GO:0032944 | regulation of mononuclear cell proliferation | 10 | 0.78 | 1.88 | 0.03 |
| GO:0045934 | negative regulation of nucleobase-containing compound metabolic process | 10 | 0.77 | 1.87 | 0.03 |
| GO:0051253 | negative regulation of RNA metabolic process | 10 | 0.77 | 1.87 | 0.03 |
| GO:1902679 | negative regulation of RNA biosynthetic process | 10 | 0.77 | 1.87 | 0.03 |
| GO:1902533 | positive regulation of intracellular signal transduction | 37 | 0.55 | 1.85 | 0.03 |
| GO:0006638 | neutral lipid metabolic process | 15 | -0.64 | -1.95 | 0.03 |
| GO:0006639 | acylglycerol metabolic process | 15 | -0.64 | -1.95 | 0.03 |
| GO:0071495 | cellular response to endogenous stimulus | 45 | 0.52 | 1.80 | 0.03 |
| GO:0070887 | cellular response to chemical stimulus | 91 | 0.42 | 1.62 | 0.03 |
| GO:0051716 | cellular response to stimulus | 174 | 0.38 | 1.59 | 0.03 |
| GO:0009966 | regulation of signal transduction | 78 | 0.45 | 1.72 | 0.03 |
| GO:1902531 | regulation of intracellular signal transduction | 47 | 0.50 | 1.76 | 0.03 |
| GO:0008284 | positive regulation of cell population proliferation | 40 | 0.52 | 1.77 | 0.03 |
| GO:0070372 | regulation of ERK1 and ERK2 cascade | 17 | 0.67 | 1.87 | 0.03 |
| GO:1903039 | positive regulation of leukocyte cell-cell adhesion | 11 | 0.74 | 1.85 | 0.03 |
| GO:0051251 | positive regulation of lymphocyte activation | 10 | 0.76 | 1.84 | 0.03 |
| GO:0009719 | response to endogenous stimulus | 60 | 0.46 | 1.69 | 0.03 |
| GO:1901564 | organonitrogen compound metabolic process | 175 | -0.29 | -1.46 | 0.03 |
| GO:0006807 | nitrogen compound metabolic process | 187 | -0.28 | -1.45 | 0.03 |
| GO:0014070 | response to organic cyclic compound | 28 | 0.58 | 1.82 | 0.03 |
| GO:0060429 | epithelium development | 32 | 0.56 | 1.80 | 0.03 |
| GO:0042981 | regulation of apoptotic process | 48 | 0.49 | 1.74 | 0.03 |
| GO:0070371 | ERK1 and ERK2 cascade | 18 | 0.65 | 1.87 | 0.03 |
| GO:0007160 | cell-matrix adhesion | 21 | 0.63 | 1.85 | 0.03 |
| GO:0002009 | morphogenesis of an epithelium | 13 | 0.70 | 1.82 | 0.03 |
| GO:0048729 | tissue morphogenesis | 13 | 0.70 | 1.82 | 0.03 |
| GO:0044706 | multi-multicellular organism process | 11 | 0.73 | 1.81 | 0.03 |
| GO:0051216 | cartilage development | 11 | 0.73 | 1.80 | 0.03 |
| GO:0061448 | connective tissue development | 11 | 0.73 | 1.80 | 0.03 |
| GO:0043067 | regulation of programmed cell death | 49 | 0.49 | 1.74 | 0.03 |
| GO:0006956 | complement activation | 50 | -0.41 | -1.71 | 0.03 |
| GO:0009967 | positive regulation of signal transduction | 50 | 0.48 | 1.70 | 0.03 |
| GO:0072378 | blood coagulation, fibrin clot formation | 10 | 0.75 | 1.81 | 0.04 |
| GO:0044093 | positive regulation of molecular function | 37 | 0.52 | 1.75 | 0.04 |
| GO:0010038 | response to metal ion | 18 | 0.64 | 1.84 | 0.04 |
| GO:0016192 | vesicle-mediated transport | 57 | 0.45 | 1.64 | 0.04 |
| GO:0042744 | hydrogen peroxide catabolic process | 10 | -0.72 | -1.92 | 0.04 |
| GO:0034109 | homotypic cell-cell adhesion | 11 | 0.71 | 1.77 | 0.04 |
| GO:0010647 | positive regulation of cell communication | 53 | 0.46 | 1.65 | 0.04 |
| GO:0023056 | positive regulation of signaling | 53 | 0.46 | 1.65 | 0.04 |
| GO:0042221 | response to chemical | 130 | 0.38 | 1.55 | 0.04 |
| GO:0045785 | positive regulation of cell adhesion | 29 | 0.56 | 1.77 | 0.04 |
| GO:0018193 | peptidyl-amino acid modification | 22 | 0.60 | 1.79 | 0.04 |
| GO:0070527 | platelet aggregation | 10 | 0.73 | 1.78 | 0.04 |
| GO:0031589 | cell-substrate adhesion | 30 | 0.54 | 1.74 | 0.04 |
| GO:0032940 | secretion by cell | 18 | 0.63 | 1.79 | 0.04 |
| GO:1902074 | response to salt | 14 | 0.66 | 1.77 | 0.04 |
| GO:0048870 | cell motility | 82 | 0.42 | 1.60 | 0.05 |
| GO:0051641 | cellular localization | 59 | 0.44 | 1.61 | 0.05 |
| GO:0071356 | cellular response to tumor necrosis factor | 10 | 0.73 | 1.76 | 0.05 |
| GO:0140352 | export from cell | 19 | 0.62 | 1.78 | 0.05 |
| GO:0018130 | heterocycle biosynthetic process | 34 | 0.52 | 1.72 | 0.05 |
| GO:0007166 | cell surface receptor signaling pathway | 94 | 0.40 | 1.56 | 0.05 |
| GO:0010467 | gene expression | 146 | 0.36 | 1.50 | 0.05 |
| GO:0032501 | multicellular organismal process | 218 | 0.34 | 1.45 | 0.05 |
| GO:0009914 | hormone transport | 10 | 0.72 | 1.75 | 0.05 |
| GO:0007159 | leukocyte cell-cell adhesion | 22 | 0.58 | 1.75 | 0.05 |
| GO:0006869 | lipid transport | 31 | -0.46 | -1.70 | 0.05 |
| GO:0010876 | lipid localization | 34 | -0.45 | -1.70 | 0.05 |
| GO:0003008 | system process | 54 | 0.44 | 1.60 | 0.05 |
| GO:0016477 | cell migration | 77 | 0.41 | 1.57 | 0.05 |
| GO:0002253 | activation of immune response | 61 | -0.36 | -1.55 | 0.05 |
| GO:0009059 | macromolecule biosynthetic process | 149 | 0.36 | 1.50 | 0.05 |
| GO:0032502 | developmental process | 154 | 0.36 | 1.48 | 0.05 |
| GO:1901576 | organic substance biosynthetic process | 161 | 0.36 | 1.48 | 0.05 |
| GO:0007565 | female pregnancy | 10 | 0.72 | 1.73 | 0.05 |
| GO:0044703 | multi-organism reproductive process | 10 | 0.72 | 1.73 | 0.05 |
| GO:0048519 | negative regulation of biological process | 156 | 0.36 | 1.48 | 0.05 |
| GO:0048856 | anatomical structure development | 149 | 0.36 | 1.49 | 0.05 |
| GO:0072376 | protein activation cascade | 11 | 0.69 | 1.72 | 0.05 |
| GO:0051239 | regulation of multicellular organismal process | 128 | 0.37 | 1.50 | 0.06 |
| GO:0042886 | amide transport | 10 | 0.71 | 1.72 | 0.06 |
| GO:0046903 | secretion | 20 | 0.59 | 1.72 | 0.06 |
| GO:0001775 | cell activation | 46 | 0.46 | 1.61 | 0.06 |
| GO:0034612 | response to tumor necrosis factor | 11 | 0.69 | 1.71 | 0.06 |
| GO:0044057 | regulation of system process | 17 | 0.61 | 1.71 | 0.07 |
| GO:0097190 | apoptotic signaling pathway | 18 | 0.60 | 1.71 | 0.07 |
| GO:2001233 | regulation of apoptotic signaling pathway | 18 | 0.60 | 1.71 | 0.07 |
| GO:0070482 | response to oxygen levels | 17 | 0.61 | 1.71 | 0.07 |
| GO:0007596 | blood coagulation | 50 | -0.37 | -1.53 | 0.07 |
| GO:0007599 | hemostasis | 50 | -0.37 | -1.53 | 0.07 |
| GO:0050817 | coagulation | 50 | -0.37 | -1.53 | 0.07 |
| GO:0050878 | regulation of body fluid levels | 50 | -0.37 | -1.53 | 0.07 |
| GO:0009636 | response to toxic substance | 22 | -0.51 | -1.71 | 0.07 |
| GO:1903037 | regulation of leukocyte cell-cell adhesion | 15 | 0.62 | 1.68 | 0.07 |
| GO:0034654 | nucleobase-containing compound biosynthetic process | 33 | 0.50 | 1.63 | 0.07 |
| GO:1901362 | organic cyclic compound biosynthetic process | 38 | 0.48 | 1.62 | 0.08 |
| GO:0071407 | cellular response to organic cyclic compound | 13 | 0.64 | 1.68 | 0.08 |
| GO:0019438 | aromatic compound biosynthetic process | 34 | 0.49 | 1.62 | 0.08 |
| GO:0009725 | response to hormone | 36 | 0.48 | 1.61 | 0.08 |
| GO:0016043 | cellular component organization | 132 | 0.35 | 1.45 | 0.08 |
| GO:0071840 | cellular component organization or biogenesis | 132 | 0.35 | 1.45 | 0.08 |
| GO:0006950 | response to stress | 204 | 0.33 | 1.41 | 0.08 |
| GO:0050789 | regulation of biological process | 276 | 0.33 | 1.40 | 0.08 |
| GO:0033993 | response to lipid | 44 | 0.46 | 1.58 | 0.08 |
| GO:0048518 | positive regulation of biological process | 198 | 0.33 | 1.41 | 0.08 |
| GO:0055088 | lipid homeostasis | 14 | -0.59 | -1.74 | 0.08 |
| GO:0009612 | response to mechanical stimulus | 10 | 0.69 | 1.66 | 0.08 |
| GO:0061061 | muscle structure development | 10 | 0.69 | 1.66 | 0.08 |
| GO:0043069 | negative regulation of programmed cell death | 31 | 0.50 | 1.62 | 0.08 |
| GO:0042742 | defense response to bacterium | 41 | 0.46 | 1.59 | 0.08 |
| GO:0009617 | response to bacterium | 57 | 0.42 | 1.52 | 0.08 |
| GO:0006508 | proteolysis | 94 | -0.31 | -1.44 | 0.08 |
| GO:0042743 | hydrogen peroxide metabolic process | 11 | -0.63 | -1.75 | 0.08 |
| GO:0009968 | negative regulation of signal transduction | 38 | 0.47 | 1.59 | 0.08 |
| GO:0009058 | biosynthetic process | 165 | 0.34 | 1.42 | 0.08 |
| GO:0065007 | biological regulation | 288 | 0.33 | 1.38 | 0.08 |
| GO:0043085 | positive regulation of catalytic activity | 28 | 0.51 | 1.62 | 0.08 |
| GO:0043066 | negative regulation of apoptotic process | 30 | 0.50 | 1.62 | 0.08 |
| GO:0044249 | cellular biosynthetic process | 161 | 0.34 | 1.42 | 0.08 |
| GO:0009987 | cellular process | 341 | 0.34 | 1.41 | 0.08 |
| GO:0019538 | protein metabolic process | 150 | -0.27 | -1.35 | 0.08 |
| GO:2001141 | regulation of RNA biosynthetic process | 31 | 0.50 | 1.60 | 0.08 |
| GO:0010648 | negative regulation of cell communication | 39 | 0.47 | 1.57 | 0.08 |
| GO:0048545 | response to steroid hormone | 15 | 0.60 | 1.64 | 0.08 |
| GO:0001666 | response to hypoxia | 16 | 0.59 | 1.63 | 0.08 |
| GO:0036293 | response to decreased oxygen levels | 16 | 0.59 | 1.63 | 0.08 |
| GO:0008152 | metabolic process | 280 | -0.26 | -1.31 | 0.08 |
| GO:1901700 | response to oxygen-containing compound | 73 | 0.39 | 1.48 | 0.09 |
| GO:0002696 | positive regulation of leukocyte activation | 12 | 0.64 | 1.64 | 0.09 |
| GO:0006355 | regulation of DNA-templated transcription | 30 | 0.50 | 1.60 | 0.09 |
| GO:0097305 | response to alcohol | 13 | 0.63 | 1.64 | 0.09 |
| GO:0097237 | cellular response to toxic substance | 19 | -0.51 | -1.65 | 0.09 |
| GO:0098754 | detoxification | 19 | -0.51 | -1.65 | 0.09 |
| GO:0098869 | cellular oxidant detoxification | 19 | -0.51 | -1.65 | 0.09 |
| GO:1990748 | cellular detoxification | 19 | -0.51 | -1.65 | 0.09 |
| GO:0032774 | RNA biosynthetic process | 32 | 0.49 | 1.58 | 0.09 |
| GO:0060627 | regulation of vesicle-mediated transport | 35 | 0.47 | 1.57 | 0.09 |
| GO:0009892 | negative regulation of metabolic process | 70 | -0.32 | -1.42 | 0.09 |
| GO:0006351 | DNA-templated transcription | 31 | 0.49 | 1.58 | 0.09 |
| GO:0051252 | regulation of RNA metabolic process | 32 | 0.48 | 1.57 | 0.09 |
| GO:0007167 | enzyme-linked receptor protein signaling pathway | 35 | 0.47 | 1.56 | 0.09 |
| GO:0048513 | animal organ development | 76 | 0.38 | 1.46 | 0.09 |
| GO:0010817 | regulation of hormone levels | 13 | 0.62 | 1.62 | 0.099 |
| GO:0032943 | mononuclear cell proliferation | 12 | 0.63 | 1.61 | 0.099 |

Terms with FDR < 0.10 were displyed.

Abbreviations: FDR, false discovery rate; NES, Normalized Enrichment Score.

**Table S10. Significantly enriched GO Biological Process pathways between ARDS subphenotypes after excluding proteins that are nominally significantly correlated with IL-6 and sTNFR1**

| ID | Description | Set size | Enrichment score | NES | FDR |
| --- | --- | --- | --- | --- | --- |
| GO:0051702 | biological process involved in interaction with symbiont | 12 | -0.764 | -2.046 | 0.05 |
| GO:0002694 | regulation of leukocyte activation | 11 | -0.749 | -1.947 | 0.05 |
| GO:0031640 | killing of cells of another organism | 11 | -0.738 | -1.920 | 0.05 |
| GO:0141060 | disruption of anatomical structure in another organism | 11 | -0.738 | -1.920 | 0.05 |
| GO:0141061 | disruption of cell in another organism | 11 | -0.738 | -1.920 | 0.05 |
| GO:0051873 | killing by host of symbiont cells | 10 | -0.764 | -1.917 | 0.05 |
| GO:0046903 | secretion | 12 | 0.765 | 1.913 | 0.05 |
| GO:0044057 | regulation of system process | 11 | 0.773 | 1.893 | 0.05 |
| GO:0051604 | protein maturation | 20 | 0.646 | 1.890 | 0.05 |
| GO:0006954 | inflammatory response | 35 | -0.536 | -1.888 | 0.05 |
| GO:0098609 | cell-cell adhesion | 28 | 0.595 | 1.872 | 0.05 |
| GO:0032940 | secretion by cell | 10 | 0.775 | 1.863 | 0.05 |
| GO:0022407 | regulation of cell-cell adhesion | 12 | 0.744 | 1.862 | 0.05 |
| GO:0002682 | regulation of immune system process | 66 | -0.443 | -1.780 | 0.05 |
| GO:0007267 | cell-cell signaling | 15 | 0.702 | 1.854 | 0.06 |
| GO:0140352 | export from cell | 11 | 0.751 | 1.839 | 0.06 |
| GO:0045184 | establishment of protein localization | 22 | 0.618 | 1.861 | 0.07 |
| GO:0060627 | regulation of vesicle-mediated transport | 19 | 0.632 | 1.811 | 0.07 |
| GO:0060341 | regulation of cellular localization | 18 | 0.631 | 1.779 | 0.07 |
| GO:0002250 | adaptive immune response | 90 | 0.419 | 1.634 | 0.07 |
| GO:0006953 | acute-phase response | 12 | -0.695 | -1.860 | 0.08 |
| GO:0032880 | regulation of protein localization | 17 | 0.636 | 1.755 | 0.08 |
| GO:0015031 | protein transport | 20 | 0.618 | 1.807 | 0.08 |
| GO:0010038 | response to metal ion | 13 | 0.698 | 1.790 | 0.08 |
| GO:0050865 | regulation of cell activation | 15 | -0.626 | -1.774 | 0.08 |
| GO:0001667 | ameboidal-type cell migration | 16 | -0.619 | -1.773 | 0.08 |
| GO:0003008 | system process | 31 | 0.551 | 1.769 | 0.08 |
| GO:0010631 | epithelial cell migration | 14 | -0.638 | -1.765 | 0.08 |
| GO:0090130 | tissue migration | 14 | -0.638 | -1.765 | 0.08 |
| GO:0090132 | epithelium migration | 14 | -0.638 | -1.765 | 0.08 |
| GO:0008015 | blood circulation | 13 | 0.687 | 1.762 | 0.08 |
| GO:0065003 | protein-containing complex assembly | 23 | 0.577 | 1.759 | 0.08 |
| GO:0001816 | cytokine production | 20 | -0.583 | -1.759 | 0.08 |
| GO:0001817 | regulation of cytokine production | 20 | -0.583 | -1.759 | 0.08 |
| GO:0003013 | circulatory system process | 16 | 0.642 | 1.721 | 0.08 |
| GO:0044403 | biological process involved in symbiotic interaction | 23 | -0.547 | -1.715 | 0.08 |
| GO:0051641 | cellular localization | 36 | 0.515 | 1.711 | 0.08 |
| GO:0008104 | protein localization | 29 | 0.532 | 1.691 | 0.08 |
| GO:0070727 | cellular macromolecule localization | 29 | 0.532 | 1.691 | 0.08 |
| GO:0022607 | cellular component assembly | 31 | 0.525 | 1.685 | 0.08 |
| GO:0044085 | cellular component biogenesis | 31 | 0.525 | 1.685 | 0.08 |
| GO:0050793 | regulation of developmental process | 44 | -0.447 | -1.653 | 0.08 |
| GO:0060284 | regulation of cell development | 10 | -0.700 | -1.755 | 0.08 |
| GO:0051223 | regulation of protein transport | 10 | 0.698 | 1.680 | 0.08 |
| GO:1901576 | organic substance biosynthetic process | 91 | 0.391 | 1.528 | 0.08 |
| GO:0009892 | negative regulation of metabolic process | 33 | -0.481 | -1.672 | 0.09 |
| GO:0070201 | regulation of establishment of protein localization | 11 | 0.688 | 1.686 | 0.09 |
| GO:0072593 | reactive oxygen species metabolic process | 10 | -0.677 | -1.698 | 0.09 |
| GO:0030168 | platelet activation | 14 | 0.647 | 1.698 | 0.09 |
| GO:0002526 | acute inflammatory response | 14 | -0.612 | -1.693 | 0.09 |
| GO:1900047 | negative regulation of hemostasis | 11 | 0.684 | 1.676 | 0.09 |
| GO:0045321 | leukocyte activation | 17 | -0.577 | -1.667 | 0.09 |
| GO:0043067 | regulation of programmed cell death | 28 | 0.524 | 1.650 | 0.09 |
| GO:0009890 | negative regulation of biosynthetic process | 19 | -0.552 | -1.647 | 0.09 |
| GO:0010558 | negative regulation of macromolecule biosynthetic process | 19 | -0.552 | -1.647 | 0.09 |
| GO:0031327 | negative regulation of cellular biosynthetic process | 19 | -0.552 | -1.647 | 0.09 |
| GO:2000026 | regulation of multicellular organismal development | 28 | -0.494 | -1.642 | 0.09 |
| GO:0043933 | protein-containing complex organization | 29 | 0.516 | 1.640 | 0.09 |
| GO:0031324 | negative regulation of cellular metabolic process | 24 | -0.504 | -1.601 | 0.09 |
| GO:0002684 | positive regulation of immune system process | 49 | -0.403 | -1.515 | 0.09 |
| GO:0051094 | positive regulation of developmental process | 21 | -0.543 | -1.667 | 0.09 |
| GO:0044249 | cellular biosynthetic process | 92 | 0.379 | 1.489 | 0.09 |
| GO:0031638 | zymogen activation | 10 | 0.678 | 1.631 | 0.09 |
| GO:0010605 | negative regulation of macromolecule metabolic process | 32 | -0.468 | -1.622 | 0.096 |
| GO:0008152 | metabolic process | 158 | -0.316 | -1.385 | 0.096 |

Terms with FDR < 0.10 were displyed.

Abbreviations: FDR, false discovery rate; NES, Normalized Enrichment Score.

**Figure S1. Descriptive concordance of protein-abundance changes in the hyperinflammatory and hypoinflammatory ARDS subphenotypes relative to healthy controls**

Each point represents one of the 437 evaluated proteins. The x- and y-axes show the log2 fold changes for the hyperinflammatory-versus-control and hypoinflammatory-versus-control comparisons, respectively. Blue points indicate proteins with concordant directions across the hyperinflammatory-versus-control, hypoinflammatory-versus-control, and pooled ARDS-versus-control comparisons; gray points indicate discordant directions. The dashed line indicates equality of the two subphenotype-versus-control log2 fold changes.

Abbreviations: ARDS, acute respiratory distress syndrome.
